# High Energy and Power Density Peptidoglycan Muscles through Super‐Viscous Nanoconfined Water

**DOI:** 10.1002/advs.202104697

**Published:** 2022-03-14

**Authors:** Haozhen Wang, Zhi‐Lun Liu, Jianpei Lao, Sheng Zhang, Rinat Abzalimov, Tong Wang, Xi Chen

**Affiliations:** ^1^ Advanced Science Research Center (ASRC) The City University of New York 85 St. Nicholas Terrace New York NY 10031 USA; ^2^ PhD Program in Physics The Graduate Center of the City University of New York 365 5th Ave. New York NY 10016 USA; ^3^ Department of Chemical Engineering The City College of New York 275 Convent Ave. New York NY 10031 USA; ^4^ PhD Program in Chemistry The Graduate Center of the City University of New York 365 5th Ave. New York NY 10016 USA

**Keywords:** actuators, artificial muscles, biomaterials, energy/power densities, nanoconfined water, water‐responsive materials

## Abstract

Water‐responsive (WR) materials that reversibly deform in response to humidity changes show great potential for developing muscle‐like actuators for miniature and biomimetic robotics. Here, it is presented that *Bacillus (B.) subtilis’* peptidoglycan (PG) exhibits WR actuation energy and power densities reaching 72.6 MJ m^−3^ and 9.1 MW m^−3^, respectively, orders of magnitude higher than those of frequently used actuators, such as piezoelectric actuators and dielectric elastomers. PG can deform as much as 27.2% within 110 ms, and its actuation pressure reaches ≈354.6 MPa. Surprisingly, PG exhibits an energy conversion efficiency of ≈66.8%, which can be attributed to its super‐viscous nanoconfined water that efficiently translates the movement of water molecules to PG's mechanical deformation. Using PG, WR composites that can be integrated into a range of engineering structures are developed, including a robotic gripper and linear actuators, which illustrate the possibilities of using PG as building blocks for high‐efficiency WR actuators.

## Graphical abstract

*B. subtilis’* PG exhibits WR actuation energy and power densities of 72.6 MJ m^−3^ and 9.1 MW m^−3^, surpassing those of existing actuators/muscles. Using PG, WR composites that can be integrated into a range of engineering structures are developed, showing strategies of using high‐performance WR actuators to perform programmed tasks under ambient conditions.

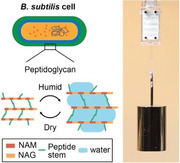

## 1 Introduction

Despite more than a century of research, mechanical actuators, which typically transduce electrical fields,^[^ ^1^ ^]^ chemical energy,^[^ ^2^ ^]^ heat,^[^ ^3^, ^4^ ^]^ and pressurized gas/liquid^[^ ^5^ ^]^ into motions, still cannot simultaneously outperform biological muscles in important metrics, including dexterity, compatibility, power density, and efficiency. These actuator challenges remain major bottlenecks for bioinspired robotic systems, especially those with small scales, to reach the performance shown in animals.^[^ ^6^ ^]^ Different from conventional actuating stimuli, the chemical potential of water drives water‐responsive (WR) materials’ swelling and shrinking when there are changes in relative humidity (RH) or water gradient. Recent demonstrations and theoretical predictions showed that such WR actuation could be extremely powerful and efficient,^[^ ^7^, ^8^, ^9^ ^]^ inspiring the growing studies and development of WR structures for actuators and artificial muscles.^[^ ^10^, ^11^, ^12^, ^13^, ^14^, ^15^, ^16^, ^17^ ^]^

Notable WR examples include a titanium oxide film^[^ ^18^ ^]^ and a twisted carbon nanotube yarn,^[^ ^19^ ^]^ which exhibit WR energy densities of ≈1250 and 1800 kJ m^−3^ (2.17 kJ kg^−1^), respectively. Microrobots equipped with WR actuators of *π*‐stacked carbon nitride films^[^ ^8^ ^]^ and polyethylene oxide nanofibers^[^ ^20^ ^]^ have been demonstrated to exhibit autonomous locomotion powered by fluctuations in ambient RH. In addition to these synthetic structures, WR materials widely exist in nature, where cellulosic structures drive many plants’ essential movements such that pinecone scales open and disperse seeds when the local environment is dry.^[^ ^21^, ^22^ ^]^ Many animal fibers and microbes are also found to show high WR performance.^[^ ^11^, ^13^, ^23^, ^24^ ^]^ For example, spider dragline silk's and regenerated *Bombyx mori* silk's WR energy densities reach 500–1600 kJ m^−3^.^[^ ^11^, ^25^, ^26^ ^]^ Prior studies have demonstrated that bacterial spores exhibit a record‐high WR actuation energy density of 21.3 MJ m^−3^, which is about 2000‐folds higher than that of mammalian muscle.^[^ ^27^ ^]^ The outstanding performance and high availability of these natural WR materials, together with the ubiquitous energy source of WR actuation, provide new opportunities for many engineering systems. For example, WR pollen papers have been used to drive a walking robot,^[^ ^28^ ^]^ and biohybrid films made of *Escherichia coli* cells have been fabricated into sweat‐responsive wearables to regulate body conditions.^[^ ^13^ ^]^ Moreover, our pioneering research has shown the possibility of using WR spores to directly and continuously harvest energy from water evaporation and subsequently using the energy for mechanical motions and electricity generation.^[^ ^29^ ^]^ However, these applications are currently hindered by unclear capabilities of these natural materials’ WR actuation, limited knowledge of energy conversion and transfer mechanisms, and the lack of practical implementation strategies for engineering systems.

**Figure 1:**
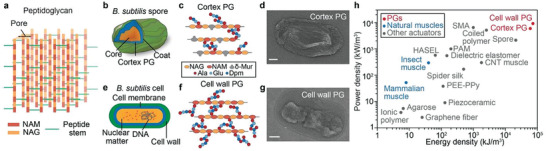
a) PG, which consists of glycan chains cross‐linked by amphiphilic peptide stems, has a three‐dimentional (3D) mesh‐like structure. b) A *B*. *subtilis* spore is mainly composed of a core with genetic information and two concentric shells, including a cortex layer of loosely cross‐linked PG (cortex PG), and a coat layer for chemical resistance. c) Cortex PG consists of NAG, NAM, and *δ*‐Mur glycan chains that are loosely cross‐linked by peptide stems of Ala‐Glu‐Dpm‐Ala. d) A SEM image of cortex PG. Scale bar in (d), 200 nm. e) PG is the main component of the *B*. *subtilis* vegetative cell wall. Cell wall PG has a similar chemical structure to that of cortex PG. f) However, its glycan chains only consist of NAG and NAM that are highly cross‐linked by similar peptide stems. g) A SEM image of cell wall PG. Scale bar in (g), 200 nm. h) PG's energy and power densities are higher than those of known muscles and stimuli‐responsive materials, including ionic polymers,^[^ ^37^ ^]^ graphene fibers,^[^ ^52^ ^]^ agarose,^[^ ^53^ ^]^ piezoceramics,^[^ ^54^ ^]^ PEE‐PPy,^[^ ^7^ ^]^ mammalian muscles,^[^ ^37^ ^]^ insect muscles,^[^ ^37^ ^]^ spider silks,^[^ ^25^ ^]^ carbon nanotube (CNT) muscles,^[^ ^19^ ^]^ dielectric elastomers,^[^ ^37^ ^]^ hydraulically amplified self‐healing electrostatics (HASEL),^[^ ^55^ ^]^ pneumatic artificial muscles (PAM),^[^ ^56^ ^]^ coiled polymers,^[^ ^57^ ^]^ shape memory alloys (SMA),^[^ ^37^ ^]^ and spores.^[^ ^9^ ^]^

Here, we report that *B.subtilis’* PG, which possesses a rigid and hierarchical structure that consists of glycan chains cross‐linked by amphiphilic peptide stems (**Figure** **1**a–g), exhibits unprecedentedly high WR energy/power densities and efficiency. When responding to RH changes, PG rapidly expands and contracts (≈0.1 s), and its WR energy and power densities reach 72.6 MJ m^−3^ (55.8 kJ kg^−1^) and 9.1 MW m^−3^ (7.0 kW kg^−1^), respectively, surpassing those of existing actuators/muscles (Figure 1h). Considering the water exchange during PG's WR actuation, we estimated PG's energy conversion efficiency to be 66.8%, surpassing those of all known natural muscles. We found that PG's powerful and efficient water‐responsiveness could be facilitated by PG's super‐viscous nanoconfined water (viscosity reaches ≈16.4 Pa∙s) and PG's stiff, deformable supramolecular structures. To demonstrate PG's potential as actuating components for miniature robotics, we developed PG/adhesive composites that actuate various mechanical structures, including a micrometer‐scale glass fiber and millimeter‐scale polymer films. Using these PG/adhesive‐based actuators, we created centimeter‐scale engineering systems, including linear actuators that can lift weights ≈3000 times heavier than the PG/adhesive composites used in the systems. These proof‐of‐concept demonstrations, together with PG's availability, low cost, and long lifetime, illustrate the great potential of using PG‐based WR actuators to power practical applications.

## 2 Results and Discussion

**Figure 2:**
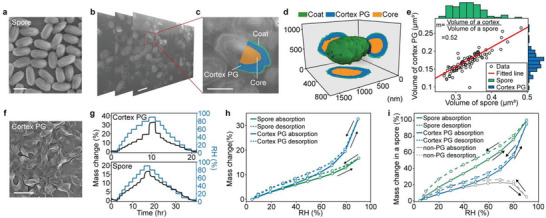
a) A SEM image of individual spores. Scale bar, 1 µm. b) Serial cross‐sectional SEM images of spores, c) showing the spatial distribution of a spore's major layers, d) were reconstructed into a 3D map. Scale bar in (b), 1 µm and in (c), 500 nm. e) A scattered plot of volumes of spores and their cortex PG layers shows that cortex PG takes up 52.4% of a spore's volume. Volumetric histograms of spores and their cortex PG are on the top and side, respectively. f) A SEM image of isolated cortex PG. Scale bar, 2 µm. g) The DVS measurements show the mass change of spores and cortex PG at various RHs during hydration and dehydration processes, h) giving their water sorption isotherms. i) Water sorption of cortex PG and non‐PG components are weighted by their mass ratios in a spore. The results indicate that cortex PG dominates spore's water uptake between 40% RH and 90% RH. The largest standard error is less than 0.64% calculated from three measurements.

PG is an essential structural component in most bacteria and their dormant spores. The discovery of PG's water‐responsiveness was inspired by *B. subtilis* spores—one of the best‐performing WR materials.^[^ ^9^ ^]^ While exploring WR mechanisms of *B. subtilis* spores, we speculated that spores’ substantial water‐responsiveness originates from a supramolecular component—PG, evidenced by PG's high water content.^[^ ^30^, ^31^ ^]^ Spores of *B. subtilis* have concentric shells, including a core that contains the genetic information, a cortex layer of loosely cross‐linked PG (cortex PG), and a coat layer that is important for spores’ chemical resistance (Figure 1b).^[^ ^32^ ^]^ Cortex PG, which is composed of N‐acetylglucosamine (NAG), N‐acetylmuramic acid (NAM), and Muramic‐*δ*‐lactam (δ‐Mur) glycan chains that are cross‐linked by peptide stems of alanine (Ala)‐glutamic acid (Glu)‐meso‐diaminopimelic acid (Dpm)‐Ala, exhibits a hierarchical and thin‐sheet structure (Figure 1b–d). To investigate the role of cortex PG in spores’ water‐responsiveness, we first analyzed cortex PG's spatial distributions within spores by taking serial cross‐sectional SEM images of spores, and then reconstructed these cross‐sectional images into a 3D map (**Figure** **2**a–d; Figure S1, Movie S1, Supporting Information). Despite the non‐uniform thickness (≈122.4 nm) (Figure 2c,d), cortex PG occupies ≈52.4% of a spore's volume, which is highly consistent across spores with various sizes (Figure 2e). Using several protein denaturing agents (see Experimental Section), we removed non‐PG components in spores, and isolated cortex PG whose geometry and chemistry were subsequently examined by a SEM and a liquid chromatography‐electrospray ionization‐mass spectrometry (LC‐ESI‐MS), respectively (Figure 2f; Figures S2,S3, Supporting Information). To understand the role of cortex PG in spores’ water exchange, a dynamic vapor sorption (DVS) system was used to measure water sorption isotherms of both spores and isolated cortex PG over an RH range from 5% to 90% (Figure 2g and Experimental Section). During hydration and dehydration cycles, spores and the isolated cortex PG can reversibly absorb and desorb 16.8 and 33.0 wt% of water, respectively (Figure 2h; Figure S4, Supporting Information). To correlate cortex PG's water uptake to that of a spore, we weighted PG's water uptake by considering cortex PG's volume ratio in a spore, and found that cortex PG's water sorption approximately contributes to 50% of that of a spore from 5% RH to 40% RH, and that cortex PG starts to dominate spore's water sorption when RH is higher than 40% (contributing ≈94% at 90% RH) (Figure 2i; Figure S5, Supporting Information). We also note that, when RH increases from 70% to 90%, spores’ non‐PG components show an unexpected water releasing phenomenon (Figure 2i).

**Figure 3:**
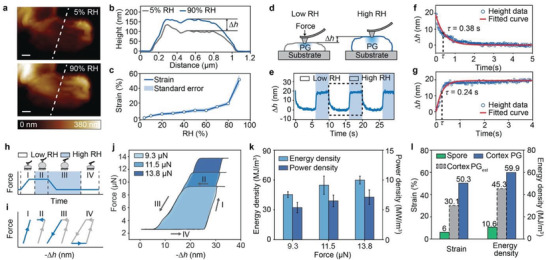
a) AFM topography images show that cortex PG expands when local RH increases from 5% to 90%. Scale bar, 200 nm. b) Cross‐sectional height profiles of cortex PG at 5% RH (grey) and 90% RH (blue). c) Cortex PG’ WR strain over RH levels. Standard errors are calculated from five measurements. d,e) The dynamic height change (Δ*h*) of a 70.6 nm thick (at 5% RH) cortex PG, when responding to alternating RH changes between 5% and 90%, shows fast WR speeds. f,g) The relaxation time constants for cortex PG's WR contraction and expansion are 0.38 and 0.24 s, respectively. h,i) A thermodynamic cycle, consisting of four stages with varying applied forces and RHs, is created by an environmental‐controlled AFM, and is used to probe cortex PG's work and power output during hydration/dehydration processes. j) Cortex PG's *F* versus −Δ*h* curves during the thermodynamic cycles with various applied forces and cycle times (8–12 s). k) Measured energy and power densities of cortex PG with different applied forces. Error bars represent standard errors calculated from five measurements. l) We predict the strain and energy density of cortex PG by assuming that non‐PG components show negligible water‐responsiveness. Cortex PG_est_ represents the estimated data.

To directly probe cortex PG's water‐responsiveness, we customized an atomic force microscope (AFM), where the local RH can be controlled while we monitor cortex PG's WR deformation and actuation force simultaneously (see Experimental Section). Using this environmental‐controlled AFM, we found that cortex PG dramatically and reversibly expands and shrinks in response to RH changes (**Figure** **3**a,b). When RH gradually increases from 5% to 90% (cortex PG reaches its equilibrium states at each RH), cortex PG shows a height change of 50.1% and a volume change of 65.2% (Figure 3c, Experimental Section; Figure S6, Supporting Information). We note that cortex PG's height increases approximately linearly with increasing RH up to 80%, and changes abruptly between 80% RH and 90% RH, which coincides with the trend of cortex PG's water sorption isotherms characterized by the DVS (Figure 2h; Table S1, Supporting Information). We also found that cortex PG's WR actuation is extremely fast (Figure 3d–g and Experimental Section); for instance, it takes ≈0.38 s (the relaxation time constant) to desorb water and contract (Figure 3f and Experimental Section) and ≈0.24 s to absorb water and expand (Figure 3g; Figure S7, Supporting Information). The faster expansion of cortex PG could be due to its glycan backbones, making PG's amphiphiles more hydrophilic. Cortex PG's large WR strain and fast response speed, together with PG's high and RH‐dependent stiffness (Young's moduli are 4.91 GPa at 5% RH and 1.77 GPa at 90% RH; Figure S8, Supporting Information), suggest high energy and power in PG's actuation. To quantify that, we programmed the environmental‐controlled AFM, and created a thermodynamic cycle to measure cortex PG's repeatable work and power output capabilities during hydration and dehydration cycles (Figure 3h–j and Experimental Section).^[^ ^9^ ^]^ The cycle consists of four stages: I) a predetermined force is applied on the top surface of cortex PG through a spherical AFM tip at ≈5% RH; II) the local RH rapidly increases to ≈90% (RH change speed is less than 100 ms), and cortex PG expands while the AFM tip maintains its force; III) the applied force is released, allowing cortex PG to fully expand under the high RH condition; IV) the cycle is finished by decreasing RH back to 5%, allowing cortex PG to shrink to its original shape (Figure 3h,i). Note that the second step in the cycle, where PG expands against a force, can represent typical application situations, in which the external load is constant. During the thermodynamic cycles, cortex PG's height change (−Δ*h*) and the applied force (*F*) are simultaneously monitored, and the enclosed area of the force versus height change curve shows the work done by the cortex PG (Figure 3i,j). To maximize cortex PG's energy and power output, we adjusted the magnitude of the force applied on cortex PG and the duration of each cycle stage (Figure 3j, Experimental Section; Figure S9, Supporting Information). Figure 3j shows that the measured work increases with increasing forces. Cortex PG's WR energy densities were then obtained by dividing the measured work by the effective volume (Figure S10, Supporting Information). We found that, with an external force of 13.8 µN (an average pressure of 354.6 MPa), cortex PG exhibits the maximum energy density of 59.9 MJ m^−3^ (Figure 3k), which is about six times higher than that of its spore (10.6 MJ m^−3^).^[^ ^9^ ^]^ Considering the time (8–12 s) taken to finish the whole thermodynamic cycle, we estimated cortex PG's power density to be 7.1 MW m^−3^, comparable to that of state‐of‐the‐art SMA (Figure 1h).^[^ ^33^ ^]^ Cortex PG's high energy density, high WR strain, and water uptake suggest that cortex PG dominates spore's water‐responsiveness. Based on spores’ WR behaviors and cortex PG's volume ratio in spores, we also estimated cortex PG's WR strain and energy density, assuming that only cortex PG contributes to spores’ water‐responsiveness. Interestingly, the estimated WR strain and energy density of cortex PG are lower than the measured ones (Figure 3l), suggesting that non‐PG components in spores dissipate energy during hydration/dehydration processes.

**Figure 4:**
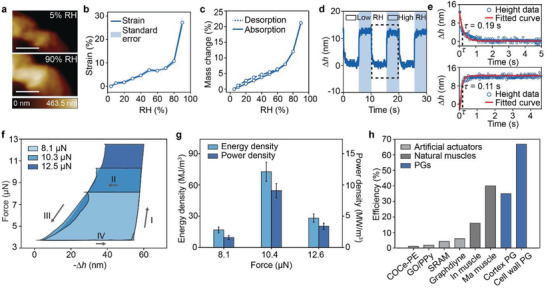
a) AFM topography images show that cell wall PG expands when RH is increased from 5% to 90%. Scale bar, 500 nm. b) Cell wall PG's WR strain over various RH levels. Standard errors are calculated from five measurements. c) Water sorption isotherms of cell wall PG. d,e) Dynamic height changes of cell wall PG with a thickness of 153 nm (at 5% RH) show fast WR speeds under changing RHs. Cell wall PG's relaxation time constants of contraction and expansion are 0.19 and 0.11 s, respectively. f) Cell wall PG's *F* versus −Δ*h* curves during thermodynamic cycles with various applied forces and cycle times (8–12 s). g) Energy and power densities of cell wall PG with different applied forces. Error bars represent standard errors calculated from five measurements. h) Energy conversion efficiencies of cortex PG, cell wall PG, and other actuators/muscles, including cyclic olefin copolymer elastomer‐polyethylene (COCe‐PE),^[^ ^38^ ^]^ graphene oxide/polypyrrole (GO/PPy) bilayer actuators,^[^ ^39^ ^]^ sheath‐run artificial muscles (SRAM),^[^ ^4^ ^]^ graphdiyne actuators,^[^ ^40^ ^]^ insect muscles (In muscle),^[^ ^58^ ^]^ and mammalian muscles (Ma muscle) (Table S2, Supporting Information).^[^ ^37^ ^]^

PG is also the main component of *B. subtilis’* vegetative bacterial cell wall with high availability (Figure 1e and Experimental Section). While cell wall PG is highly cross‐linked (cross‐linking ratios of 33% for cell wall PG and 6% for cortex PG),^[^ ^34^ ^]^ it shares similar glycan chains and peptide stems as cortex PG, and shows a similar hierarchical and stiff (Young's moduli are 4.49 GPa at 5% RH and 1.77 GPa at 90% RH) structure (Figure 1f,g; Figure S8, Supporting Information) with nanoscale pores (≈6.8–38.4 nm in diameter).^[^ ^35^ ^]^ Therefore, we speculate that *B*. *subtilis’* cell wall PG should also be WR. Using the same AFM setup, we characterized cell wall PG's surface topographies at various RH levels, and showed that cell wall PG also expands dramatically (a WR height change of 27.2% and a volume change of 45.8%) when local RH is increased from 5% to 90% (**Figure** **4**a,b, Experimental Section; Figure S11, Supporting Information). Compared to cortex PG, cell wall PG shows a smaller WR strain which directly correlates to its less water uptake of 21 wt% (Figure 4c; Figure S4, Supporting Information), potentially resulted from a denser structure owing to cell wall PG's higher cross‐linking ratio. Nonetheless, cell wall PG and cortex PG share similar strain versus RH trends and water sorption isotherms, where abrupt changes occur at ≈80% RH (Figures 2h,3c,4b,c). Cell wall PG also possesses similar WR actuation that is highly reversible and extremely fast as that of cortex PG (Figures 3e,4d; Figure S7, Supporting Information). Notably, cell wall PG shows faster dehydration (0.19 s) and hydration (0.11 s) speeds than those of cortex PG (Figure 4e), which could be due to its less water uptake.

We also measured energy and power densities of cell wall PG by using the same thermodynamic cycle (Figure 3h,i), where the applied force and duration in each stage were varied to maximize the energy and power output (Figure 4f; Figure S12, Supporting Information). Surprisingly, we found that cell wall PG's WR energy and power densities are extremely high, reaching 72.6 MJ m^−3^ and 9.1 MW m^−3^, respectively (Figure 4g, **Table** **1**). Note that the total energy input (*µ*) relies on the amount of water exchange and water's activities during hydration/dehydration processes, and it can be given by^[^ ^36^ ^]^

(1) $$\mu =nR_{i}Tln\left(\frac{a_{1}}{a_{2}}\right)$$

where *R*_i_ is the ideal gas constant (8.314 J K^−1^mol^−1^), *T* is the temperature (298.15 K), *n* is moles of exchanged water molecules between 5% RH and 90% RH, and *a*_1_ and *a*_2_ are activities of water vapor at 90% RH and 5% RH, respectively. We estimated cortex PG and cell wall PG's energy conversion efficiencies to be 35.0% and 66.8%, respectively, which are comparable to the efficiency of mammalian muscle (≈40%),^[^ ^37^ ^]^ and are much higher than those of recently reported actuators (≈1.1–6.0%) (Figure 4h; Table S2, Supporting Information).^[^ ^4^, ^38^, ^39^, ^40^ ^]^

**Table 1:** WR properties of cortex PG and cell wall PG

	Strain [%]	Stiffness [GPa]	Response speed [s]	Energy density [MJ m^−3^]	Power density [MW m^−3^]	Maximal stress [MPa]	Efficiency [%]
Cortex PG	50.1	4.91 (dry) 1.77 (humid)	0.38 (dehydration) 0.24 (hydration)	59.9	7.1	354.6	35.0
Cell wall PG	27.2	4.49 (dry) 1.77 (humid)	0.19 (dehydration) 0.11 (hydration)	72.6	9.1	205.6	66.8

We found that cell wall PG's remarkable WR performance could relate to the anomalously high viscosity of water confined in PG's stiff and deformable nanoporous structures. Using the poroelastic theory and PG's relaxation time constants,^[^ ^41^, ^42^ ^]^ we estimated pore water's viscosity to be ≈16.4 Pa∙s (see Supporting Information), ≈10^4^ times greater than that of bulk water. Such high viscosity shares similarities with our recently observed evaporation‐induced H‐bonding strengthening of water confined in WR tripeptide crystals.^[^ ^43^ ^]^ Note that the high viscosity of PG's nanoconfined water does not contradict the fast WR speed and high power density, which could be explained by PG's small size and large surface‐to‐volume ratio that facilitate water diffusion. Thermodynamically, materials’ WR actuation is driven by the chemical potential difference between water inside the material and water vapor outside. Such potential difference causes an osmotic pressure at the water/air interface, which subsequently induces a negative pressure gradient within the material's confined water, and drives a fluid flow and structural deformation until the material's elastic stress balances the negative pressure, or cavitation occurs. It is very likely that, during evaporation, the super‐viscous nanoconfined water, resulted from PG's unique nanoscale and amphiphilic pores, can resist cavitation and effectively translate the osmotic pressure to shrink PG's stiff and continuous structures that regain their original shapes upon rehydration. This hypothesis suggests that high‐performance WR materials require nano‐porosity and amphiphilicity that lead to highly viscous flow to drag pore surfaces,^[^ ^44^ ^]^ and structures with high mechanical stiffness and ductility that allow the structure to store and release a large amount of elastic energy.

**Figure 5:**
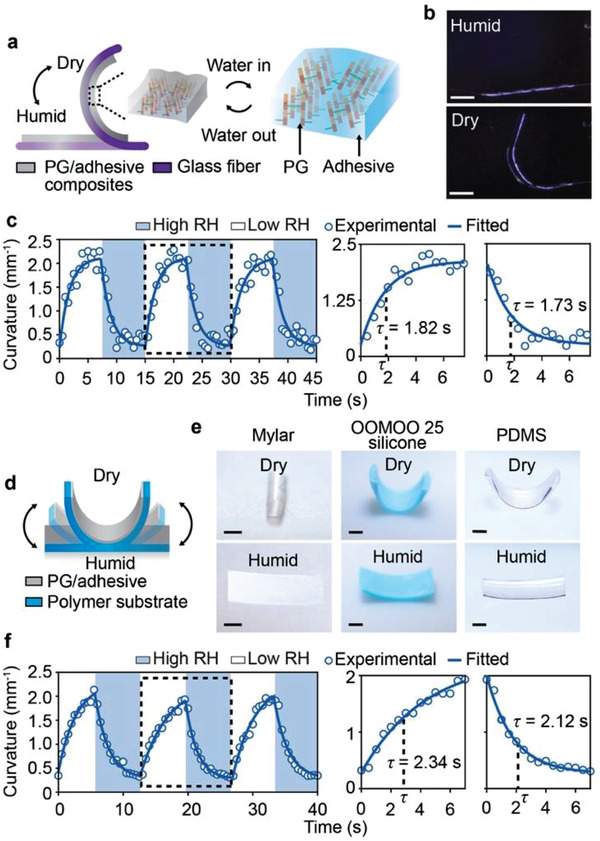
a,b) A 10.6 µm glass fiber with 11.6 µm thick PG/adhesive composites coated on one side bends under dry conditions and straightens under humid conditions actuated by the WR composites. Scale bar, 0.5 mm. c) Relaxation time constants of the PG/adhesive‐coated glass fiber are 1.82 s and 1.73 s for bending and straightening, respectively. d,e) PG/adhesive‐coated Mylar, OOMOO 25 silicone, and PDMS bend and straighten in response to changing humidity. Scale bar, 1 mm. f) The PG/adhesive‐coated Mylar has relaxation time constants of 2.34 s for bending and 2.12 s for straightening.

To demonstrate the potential of using PG as actuating components for engineering systems, we developed PG/adhesive composite muscles by simply mixing cell wall PG with a commercial adhesive (Elmer's glue). These PG/adhesive muscles (Young's modulus is 1.33 GPa at 5% RH and 0.47 GPa at 90% RH; Figure S13, Supporting Information) are highly compatible with both stiff and soft mechanical structures with sizes varying from micro‐ to macroscales. For example, a stiff (Young's modulus of 70 GPa) and microscale glass fiber (10.6 µm in diameter) with PG/adhesive composites coated on its surface rapidly bends (1.82 s) and straightens (1.73 s) due to the net force generated by the PG/adhesive composite layer when the local RH is alternated between 5% and 90% at 25 °C (**Figure** **5**a–c, Experimental Section; Movie S2, Supporting Information). During such actuation, the energy density of PG/adhesive composites reaches 4.90 MJ m^−3^ (calculated by only considering composites’ work done on the passive glass fiber, Supporting Information), which is more than two orders of magnitude higher than that of mammalian skeletal muscle (8 kJ m^−3^),^[^ ^27^ ^]^ and is also higher than those of other WR materials used for similar bending actuators.^[^ ^7^, ^10^, ^28^, ^45^, ^46^, ^47^, ^48^ ^]^ We also deposited PG/adhesive composites on soft polymers, including Mylar, OOMOO 25 silicone, and polydimethylsiloxane (PDMS), which are frequently used structural materials in soft robotics (Figure 5d,e; Figure S14, Table S3, Supporting Information). Similar to the PG/adhesive‐coated glass fibers, these bilayer structures rapidly and reversibly actuate in response to RH changes (Figure 5d–f; Figures S14,S15, Movie S3–S5, Supporting Information). For example, the PG/adhesive‐coated Mylar film can respond to RH changes within ≈3 s (Figure 5f), and bend and straighten over 180,  000 cycles without significant performance degradation (the performance degradation rate is 5.17 × 10^−5^% per cycle. Figure S16, Supporting Information). We also note that curvatures of the PG/adhesive‐coated Mylar film increase with increasing temperatures and pH, which could be caused by changes in nanoconfined water's chemical potential and H‐bonding network (Figure S17, Supporting Information).

**Figure 6:**
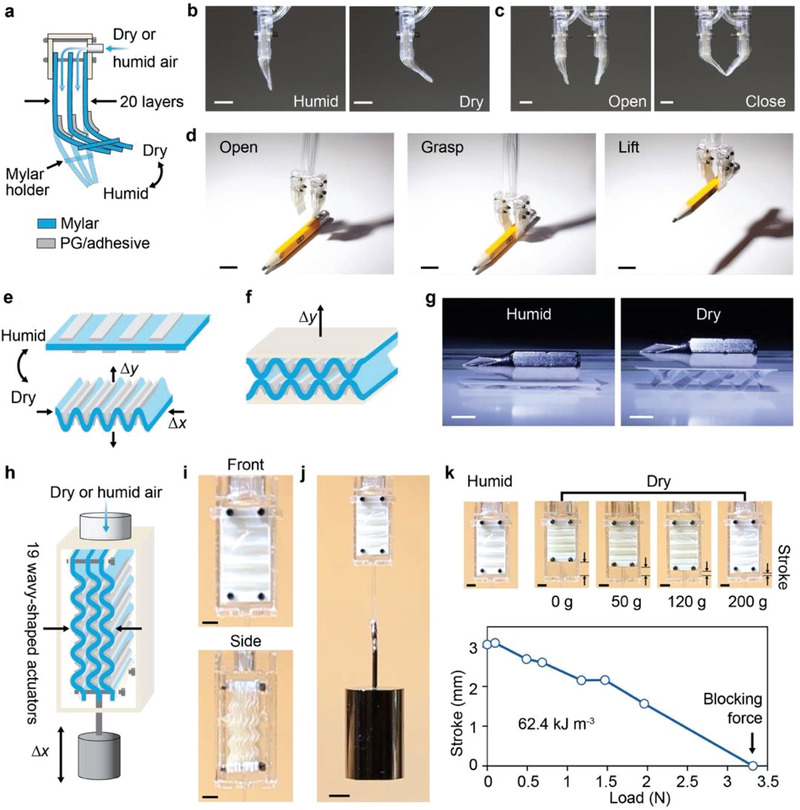
a,b) An active finger, which consists of twenty PG/adhesive‐coated Mylar films, bends and straightens programmed by an RH‐control system that locally delivers humid or dry air to PG/adhesive layers. Scale bar, 5 mm. c) A PG‐based soft gripper, consisting of two active fingers, is controlled to open and close. Scale bar, 5 mm. d) The PG‐based soft gripper grasps and lifts a pencil (2.2 g) under ambient conditions. Scale bar, 10 mm. e) A wavy‐shaped WR actuator generates linear actuation in vertical and horizontal directions. f) A push actuator, consisting of two wavy‐shaped WR actuators, g) reversibly lifts a screwdriver bit (4.7 g) in response to RH changes. Scale bar, 5 mm. h) A pull actuator consists of nineteen wavy‐shaped WR actuators. The actuation of the pull actuator can be programmed by a portable RH‐control system. i) Front and side views of the pull actuator. Scale bar, 5 mm. j,k) The pull actuator that can reversibly lift weights shows an energy density of 62.4 kJ m^−3^. Scale bar, 5 mm.

Using these soft PG‐based polymer actuators, we created origami structures that self‐adapt to changing environments that trigger their structural transformations. For instance, a precut Mylar film with patterned PG/adhesive muscles can morph into a cubic structure as pre‐condensed water droplets evaporate, when the local environment changes from a humid condition to a dry condition (Movie S6, Supporting Information). In addition to these origami structures that are passively triggered by environmental changes, we developed several strategies to demonstrate the on‐demand actuation of WR muscles for practical applications. For instance, we fabricated a PG‐based soft gripper that contains two active fingers with layered structures formed by stacking twenty PG/adhesive‐coated Mylar films (**Figure** **6**a–d; Figures S18,S19, Supporting Information). This soft gripper is equipped with a portable RH‐control system, which allows for the rapid delivery of low‐pressure air with programmed RHs to individual PG/adhesive layers through fine gaps between individual Mylar films, and thus the soft gripper's actuation will not be affected by ambient RH fluctuations (Figure 6b,c; Movie S7, Supporting Information). With such a system, the soft gripper that only contains ≈25 mg of PG/adhesive composites can perform programmed tasks, including grasping and lifting a 2.2 g pencil (Figure 6d; Movie S8, Supporting Information) and a 4.7 g screwdriver bit (Movie S9, Supporting Information).

By patterning PG/adhesive composites on both sides of a Mylar film, we also developed a wavy‐shaped linear actuator (Figure 6e) whose actuation can be scaled up to generate large forces and strokes. For example, we created a push actuator by stacking two wavy‐shaped actuators vertically (Figure 6f; Supporting Information). The push actuator shows an amplified stroke, and can reversibly raise a screwdriver bit (4.7 g) that is more than 800 times heavier than the PG/adhesive composites (5.8 mg) used in the actuator (Figure 6g; Movie S10, Supporting Information). In addition to the push actuator, we also bundled these wavy‐shaped actuators in parallel, and created a pull actuator (Figure 6h; Figure S20, Supporting Information). Equipped with a portable RH‐control system, the pull actuator with nineteen wavy‐shaped actuators exhibits a maximum strain of 13.2%, and can lift weights of 338.5 g, almost 3000 times heavier than that of the PG/adhesive composites used in the pull actuator (0.12 g) (Figure 6i–k; Movie S11,S12, Supporting Information). Using the stroke versus load curve of this pull actuator (Figure 6k), we estimated the energy density of PG/adhesive composites to be 62.4 kJ m^−3^, which is about seven times higher than that of mammalian muscle (8 kJ m^−3^).

## 3 Conclusion 

In conclusion, *B. subtilis* PG's extraordinary WR performance, including high energy/power densities and efficiency, together with PG's high durability and availability, suggest PG's great potential as a building block to develop powerful actuators and artificial muscles that could not only advance existing engineering systems, but also enable new applications. PG's forceful WR actuation sheds light on the fundamental mechanisms of evaporation‐induced deformation of WR materials. The counterintuitive observation of super‐viscous fluid flow in PG's fast and powerful actuation suggests that secondary bonds of water–water and water–structure are unexpectedly strong during PG's WR actuation. It is likely that, during dehydration, the enhanced secondary bonding could efficiently transfer tension induced by the chemical potential difference at the pore water/air interface to shrink PG's structure, and avoid cavitation. The simultaneously stiff and deformable supramolecular structure of PG allows the elastic energy to be effectively stored, and then released during rehydration. We expect that, as observed in PG, the high stiffness (≈1–5 GPa) and nanoscale pores that enhance water–structure interactions are critical to PG's WR actuation, and that they could serve as a guideline for the rational design of high‐efficiency WR structures.

We envision that this emerging category of WR actuators could offer numerous advantages over traditional actuators (Table S4, Supporting Information). For example, soft actuators usually require high‐pressure gas/liquid, high voltages/powers, or high temperatures, which largely limit their applications. WR actuators, which generate a high pressure and powerful actuation by working with low‐pressure dry and humid air, could remove design constraints and find possibilities in driving soft robots, exoskeletons, as well as miniature engineering systems. Despite these advantages, the development of WR actuators and their system‐level applications are still in the early stage. We expect that our proof‐of‐concept demonstrations that show possible strategies of using PG‐based WR actuators to perform programmed tasks under ambient conditions could inspire and promote the development of WR actuator‐based engineering systems toward real‐world applications.

## 4 Experimental Section

### Preparation of *B. Subtilis* Vegetative Cells and Spores

The growth of *B. subtilis* cells were conducted by adding 1 mL of spore suspension (BGA, Sigma Aldrich) in 200 mL BD Difco nutrient broth_(aq)_, consisting of 3.2 g of Difco powder, 0.4 g of KCl, 0.125 g of MgSO_4_∙7H_2_O, 47.2 mg of Ca(NO_3_)_2_∙4H_2_O, 0.2 g of glucose, 0.44 mg of FeSO_4_∙7H_2_O, and 1 mg of MnCl_2_. All chemicals were purchased from Fisher Scientific. The culture was incubated on a hot plate (Isotemp, Fisherbrand) at 37 °C and aerated vigorously with a magnetic stir bar. After 19 h, vegetative cells were separated from the culture by centrifuging the culture at 14 000 × *g* for 8 min.^[^ ^49^ ^]^ To grow *B*. subtilis spores, the same culture with 1 mL of spore suspension in 200 mL nutrient broth was prepared and incubated at 37 °C for 4 days. To harvest spores, the culture suspension was first centrifuged at 10 000 × *g* for 3 min to remove the nutrient broth and the pellet was resuspended in purified water (Milli‐Q), which was performed twice.^[^ ^50^ ^]^ Subsequently, the resulted mixture suspension was centrifuged at 455 × *g* for 7 min and the pellet was resuspended in purified water, which was performed five times to remove cell debris. Finally, *B. subtilis* spores were separated from their cells by centrifuging the suspension at 169 × *g* for 5 min, and purified spores were collected from the supernatant (Figure 2a).

### PG Preparation

To isolate cortex PG from *B. subtilis* spores, 55 mg of spores were added to 1 mL of protein denaturation solution, consisting of 50 mm Tris‐HCl (pH 8), 8 m urea, 35 mm sodium dodecyl sulfate (SDS), and 50 mm dithiothreitol (DTT), incubated for 2 h at 37 °C, and were pelleted. The denaturation process was repeated once. The denatured spores were washed three times with DI water, resuspended in 1 mL of 0.05 g mL^−1^ trichloroacetic acid (TCA) solution, and boiled at 95 °C for 6 min. Subsequently, the cortex PG was pelleted from denatured proteins (14 594 × *g* for 3 min) and resuspended in a 0.5 m Tris‐HCl (pH 9.5) solution to remove the TCA. The sample was then pelleted (14 594 × *g* for 3 min), resuspended in a solution consisting of 50 mm Tris‐HCl (pH 8), 105 mm SDS, and 50 mm DTT solution, and boiled for 20 min. The whole process starting from the treatment with 0.05 g mL^−1^ TCA was repeated once to remove non‐PG components in the spores completely. To isolate cell wall PG from *B. subtilis* vegetative cells, 55 mg of cells were added to 10 mL of 347 mm SDS, boiled for 3 h, and washed five times with DI water. The resulting cells were then treated with 10 mL of 2 mg mL^−1^ pronase (Sigma Aldrich) at 50 °C for 2 h, which was repeated once to isolate cell wall PG. After being washed by DI water seven times, the cell wall PG was lyophilized using a centrifugal vacuum concentrator (HyperVAC, Gyrozen). The extraction efficiency of cell wall PG was characterized to be ≈68% by comparing the mass of lyophilized cell wall PG to that of cells from the same batch. All the chemicals mentioned in this section were purchased from Fisher Scientific unless specifically noted.

### Water Sorption Isotherms

Water sorption isotherms of PGs and spores were measured by a DVS system (DVS Intrinsic, Surface Measurement Systems) at 25 °C. Samples of ≈9 mg of spores, ≈3 mg of cell wall PG (Sigma Aldrich), and ≈0.7 mg of isolated cortex PG were separately loaded in the DVS chamber for characterization. When the RH was cycled between 5% and 90%, masses of PGs and spores were measured in real‐time. To obtain water sorption isotherms over various RH levels, each RH level was set to either maintain for at least 20 min or change to the next level after the mass change rate was lower than 0.00005 mg min^−1^ for 5 min (the longer durations were chosen). For each sample, three cycles of water sorption isotherms were collected and analyzed by the DVS Control Software (Figure S4, Supporting Information).

### WR Strain Characterization

PG was deposited on a silicon wafer, and its water‐responsiveness was subsequently characterized by using an AFM (Multimode 8, Bruker) at 25 °C. The local RH near PG samples was controlled by fine‐tuning the flow rates of humid or dry air injected near the sample inside an enclosed AFM chamber until RH was stabilized at certain levels. A commercial RH sensor (HIH‐4021‐003, Honeywell) was placed near PG samples to monitor the local RH continuously. After RH was stabilized at a certain level, PG's topographies were measured by using an AFM probe with a tip radius of ≈2 nm (SCANASYST‐AIR, Bruker). To analyze PG's WR strain, PG's topographies at various RH levels were compared to those at 5% RH. PG's volumetric change was characterized by using the NanoScope Analysis (Bruker).

### WR Speed Characterization

PG's dynamic height change in response to RH changes was measured by a customized AFM system (Multimode 8, Bruker). To rapidly change RH near PG, streams that carry dry (5%) and humid (90%) air were locally delivered to a small region surrounding PG by using two microtubes, and these streams were controlled by a solenoid valve (VK332Y, SMC) with a response time of 10 ms and a LabVIEW program. The dynamic curvature changes (Γ) of PG/adhesive‐coated glass fibers and polymer substrates were characterized by using a similar RH control system, where millimeter‐scale tubes were used to deliver streams. Curvatures of PG/adhesive‐coated structures were recorded by using cameras (Axiocam ERc 5s, Zeiss; Canon EOS Rebel SL1), and the video recordings were processed by using an image processing software (Image J, NIH image). The WR speed relaxation time constants (*τ*) were quantified by fitting the plots of height (PG) or curvature changes (PG/adhesive‐coated structures) over time (*t*) with exponential decay/growth functions,^[^ ^51^ ^]^ given by:

(2) $$\Gamma \left(t\right)=\left(\Gamma_{\mathrm{Max}}-\Gamma_{\mathrm{Min}}\right)e^{-t/\tau }+\Gamma_{\mathrm{Min}}$$

(3) $$\Gamma \left(t\right)=-\left(\Gamma_{\mathrm{Max}}-\Gamma_{\mathrm{Min}}\right)e^{-t/\tau }+\Gamma_{\mathrm{Max}}$$

where Γ is the height or the curvature, Γ_Max_ and Γ_Min_ are the maximum and minimum height or curvature, respectively.

### PG's Energy/Power Densities Characterization

PG's WR energy and power densities were measured by using the customized AFM and a thermodynamic cycle that was previously used to measure spores’ energy density (Figures 3h–k,4f,g; Figures S9a,S12a, Supporting Information).^[^ ^9^ ^]^ To create the thermodynamic cycle, the RH levels were controlled using the same setup as that for PG's WR speed characterization, and an AFM probe (LRCH‐250, Team Nanotec) was used to apply forces on PG samples while monitoring the indentation depth by using a high‐speed data acquisition card (PCI‐6115, National Instruments) and an analog‐summing amplifier (SIM980, Stanford Research Systems) controlled by a LabVIEW program. To probe PG's maximum energy and power output, the magnitude of the applied force and the duration for each stage were adjusted using the LabVIEW program (Figures S9a–c,S12a–c Supporting Information). The full thermodynamic cycle was repeated at least five times for each measurement (Figures S9d–f,S12d–f, Supporting Information). The energy density was calculated by dividing the work done by the effective volume (see calculation details in Supporting Information). The power densities were calculated by considering the durations (8–12 s) of individual thermodynamic cycles.

### PG‐Based Micro‐ and Macro‐Structures

Lyophilized cell wall PG was first added in an aqueous commercial adhesive (Elmer's glue, Amazon) solution to form a composite solution that consisted of 51.98 mg mL^−1^ of PG and 17.33 mg mL^−1^ of adhesive. To coat the PG/adhesive composite on a glass fiber (TRUE COMPOSITES, Amazon), a 2.5 µL PG/adhesive solution was deposited on the glass fiber, and was allowed to dry and form an 11.6 µm thick PG/adhesive composite layer. The energy density of the PG/adhesive composites was estimated by considering the glass fiber's maximum WR curvature (Supporting Information). The PDMS film was prepared using a liquid silicone elastomer kit (Sylgard 184, Fisher Scientific). The liquid silicone elastomer base and the liquid curing agent in the kit were mixed at a mass ratio of 10:1 in a petri dish. The mixture was degassed in a vacuum chamber for 2 h, and subsequently cured at 23 °C for 12 h to form a 0.5 mm thick PDMS film. The OOMOO 25 silicone film was prepared using a Smooth‐On silicone kit (Amazon). The solution A and solution B in the kit were mixed at a mass ratio of 1:1 in a petri dish. The mixture was degassed in a vacuum chamber for 2 h, and subsequently cured at 60 °C for 2 h to form a 0.5 mm thick OOMOO 25 silicone film.

To coat these soft polymers with PG/adhesive composites, a 3.3 µL PG/adhesive solution was deposited on 3 mm × 6 mm polymer substrates, including a 12 µm thick Mylar (Premier Lab Supply) film, a 0.5 mm thick OOMOO 25 silicone film, and a 0.5 mm thick PDMS film. The depositions were allowed to dry at 4 °C at ≈90% RH, forming 8 µm thick PG/adhesive composites.

### Statistical Analysis

For statistical analyses, there was no pre‐processing of data. All experimental data are presented as mean ± SEM (standard error of mean) with *n* = 5, indicating the number of replicates unless otherwise noted. Statistical analysis was carried out using Matlab.

## Conflict of Interest

The authors declare no conflict of interest.

## Author Contribution

H.W. and Z.‐L.L. contributed equally to this work. X.C. conceived and initiated the project. Z.‐L.L., S.Z., and T.W. performed the SEM and FIB experiments. Z.‐L.L. analyzed spore's components and isolated the cortex PG. R.A. and Z.‐L.L. performed LC‐ESI‐MS measurements and data analysis. H.W., Z.‐L.L., and X.C. performed AFM and DVS measurements and data analysis. H.W., Z.‐L.L., and J.L. prepared soft PG muscles and their engineering demonstrations. All authors contributed to data analysis and discussed the results. H.W., Z.‐L.L., and X.C. wrote the paper, and X.C. supervised the project.

## Supporting information

Supporting Information

Supplemental Movie 1

Supplemental Movie 2

Supplemental Movie 3

Supplemental Movie 4

Supplemental Movie 5

Supplemental Movie 6

Supplemental Movie 7

Supplemental Movie 8

Supplemental Movie 9

Supplemental Movie 10

Supplemental Movie 11

Supplemental Movie 12

## Data Availability

The data that support the findings of this study are available from the corresponding author upon reasonable request.
