# Supplementary material for: High Energy and Power Density Peptidoglycan Muscles through Super‐Viscous Nanoconfined Water
Source: Adv Sci (Weinh). 2022 Mar 14;9(15):2104697. doi: 10.1002/advs.202104697 (PMC9130901; doi:10.1002/advs.202104697)
Supplement: Supplementary file 1 — Supporting Information [file ADVS-9-2104697-s009.pdf]

ADVANCED SCIENCE
Open Access

Supporting Information

for *Adv. Sci.*, DOI 10.1002/advs.202104697

High Energy and Power Density Peptidoglycan Muscles through Super-Viscous Nanoconfined Water

*Haozhen Wang, Zhi-Lun Liu, Jianpei Lao, Sheng Zhang, Rinat Abzalimov, Tong Wang and Xi Chen**

ADVANCED SCIENCE
Open Access

Supporting Information

for *Adv. Sci.*, DOI: 10.1002/advs.202104697

High energy and power density peptidoglycan muscles through super-viscous nanoconfined water

*Haozhen Wang, Zhi-Lun Liu, Jianpei Lao, Sheng Zhang, Rinat Abzalimov, Tong Wang, Xi Chen**

Supporting Information

High energy and power density peptidoglycan muscles through super-viscous nanoconfined water

*Haozhen Wang, Zhi-Lun Liu, Jianpei Lao, Sheng Zhang, Rinat Abzalimov, Tong Wang, Xi Chen**

H. Wang, Z.-L. Liu, J. Lao, Dr. S. Zhang, Dr. R. Abzalimov, Dr. T. Wang, Prof. X. Chen
Advanced Science Research Center (ASRC)
The City University of New York
85 St. Nicholas Terrace, New York, NY 10031, USA
E-mail: xchen@gc.cuny.edu

Z.-L. Liu, J. Lao, Prof. X. Chen
Department of Chemical Engineering
The City College of New York
275 Convent Avenue, New York, NY 10031, USA

H. Wang, Prof. X. Chen
PhD Program in Physics
The Graduate Center of the City University of New York
365 5th Avenue, New York, NY 10016, USA

Prof. X. Chen
PhD Program in Chemistry
The Graduate Center of the City University of New York
365 5th Avenue, New York, NY 10016, USA

Spatial distributions of cortex PG in spores

We cross-linked proteins and lipids in spores to preserve the geometries of spores during the invasive spatial distribution analysis. To cross-link spores' proteins, we first rinsed spore pellets for 5 min using a solution consisting of 4% glutaraldehyde and 0.1 M cacodylate buffer (pH 7.4). Spore pellets were then rinsed for 30 min using another solution consisting of 2% aqueous osmium tetroxide and 0.1 M cacodylate buffer (pH 7.4) to fix both proteins and lipids. After rinsing the pellets with DI water three times (5 min each), we subsequently removed water in spores by rinsing the sample with ethanol of increasing concentrations (30%, 40%, and 50%). To enhance the contrast of spores' components in SEM images, we soaked the sample in a solution consisting of 1% uranyl acetate and 50% ethanol for 30 min, and then rinsed with ethanol of increasing concentrations (50%, 70%, 80%, 90%, 100%, 100%, and 100%) to replace water with ethanol completely. To infiltrate the pellets with the resin solution, which has a formula of 20 mL EMBed-812, 9 mL dodenyl succinic anhydride (DDSA), and 12 mL methyl-5-norbornene-2,3-dicarboxylic anhydride (NMA), we rinsed the pellets with 100% propylene oxide (PO) for 5 min three times, and then rinsed the pellets with increasing concentration of resin (50%, 70%, and 100% resin solution in PO) for 1 hour each. We then transferred the pellets into a Beem capsule (EMS) and filled the capsule with a 1.5 mL resin solution having a formula of 20 mL EMBed-812, 9 mL DDSA, 12 mL NMA, and 700 μL 2,4,6-Tri(dimethylaminomethyl)phenol (DMP-30). The capsule was left open under vacuum for two hours to degas the resin, and then stored in an oven at 60°C for two days. All chemicals were purchased from Electron Microscopy Sciences. To expose spores for SEM imaging, we used an ultramicrotome (Ultracut E, Reichert-Jung) to slice the sample until spores were visible by an optical microscope (Stereo microscope, American Optical Corporation). A focused ion beam - scanning electron microscope system (FIB-SEM, FEI Helios) was used to mill the sample and obtain serial cross-sectional SEM images (Figure S1a). During the milling and imaging process, 422 cross-sectional images with slices thickness of 20 nm were automatically collected using Auto Slice & View software in the FIB-SEM system (Figure S1b). To create a 3D map that shows cortex PG's spatial distributions, we used a 3D reconstruction software (Avizo) to stack and align the collected images (Figure S1c). To analyze spores' components, we applied a fast Fourier transform (FTT) filter to enhance the contrast and reduce the background noise of SEM images. The coat, cortex PG, and core

were then determined based on the contrast of these images (Figure S1d-e). Using this method (Movie S1), we analyzed cortex PG's spatial distributions in 85 spores.

Identification of cortex PG's chemical structure using LC-ESI-MS

A liquid chromatography-electrospray ionization-mass spectrometry (LC-ESI-MS) was used to characterize isolated cortex PG's molecular structure (Figure S2a).[1] To hydrolyze isolated cortex PG, we incubated the cortex PG with the mutanolysin enzyme (Sigma-Aldrich) in 1 mL of 25 mM sodium phosphate (pH 5.6) at 37 °C for 15 hours and then boiled it on a hot plate for 3 minutes to stop the hydrolysis. After the solution was centrifuged at 14,000 × g for 8 min, the supernatant that contains digested cortex PG was collected and stored at -20 °C. To run the LC-ESI-MS test, we used the maXis-II UHR-ESI-QqTOF mass-spectrometry (Bruker Daltonics) coupled to an Ultimate-3000 UHPLC system (Fisher Scientific). To separate various hydrolysates by the liquid chromatography, the elution buffer gradients used were: 0-5 min isocratic start at 5% solvent B (Acetonitrile, 0.25% formic acid) and 95% solvent A (Water, 0.25% formic acid); 5-20 min gradient to 95% B; 20-25 min isocratic mode at 95% B. Our sample was introduced by single injections of 50 μL supernatant into the ZORBAX 300SB-C18 column 2.1 mm × 100 mm (Agilent) at 30 °C with a flow rate of 200 μL min^{-1}, and the sample's mass-spectral signals were detected by the MS with errors less than 3 ppm.[1] To analyze the signals, we developed an algorithm to deconvolute the detected mass-spectral peaks, where exact molecular weights of hydrolysates were calculated based on possible combinations of the glycan chains and peptide stems. In cortex PG hydrolysates, the glycan chains consist of repeating units of NAG and δ-Mur with one NAM connected to the NAG end (Figure S2a). Note that NAM connects to peptide stems consisting of Ala, Glu, and Dpm residues, and that there are only 8 possible combinations of the peptide sequences (Figure S2b-c).[2] All possible cortex PG hydrolysates' monoisotopic masses were calculated by using an equation:

$$\left(\frac{m}{z}\right) = \frac{M_0 + zA^+}{z} \quad \text{— (S1)}$$

where $\frac{m}{z}$ is the predicted mass to charge ratio of cortex PG hydrolysates, M_0 is the monoisotopic mass of PG hydrolysates, A^+ is the monovalent cation (H^+, Na^+), and z is the number of charges.

Using this method, we have identified six cortex PG hydrolysates with accuracies (ε) within 3 ppm. These identified hydrolysates include (NAG)-(NAM)-Ala-Glu-Dpm-Ala//Ala-Glu-Dpm-Ala//Ala-Glu-Dpm-Ala (ε=3.22 ppm for M_0+2H^+), (NAG)-(δ-Mur)-(NAG)-(NAM)-Ala-Glu-Dpm-Ala (ε=1.58 and 2.04 ppm for $M_0+H^++Na^+$ and M_0+2H^+, respectively), (NAG)-(δ-Mur)-(NAG)-(δ-Mur)-(NAG)-(NAM)-Ala (ε=1.99 ppm for M_0+2H^+), (NAG)-(δ-Mur)-(NAG)-(δ-Mur)-(NAG)-(NAM)-Ala-Glu-Dpm-Ala (ε=1.77 ppm for M_0+2H^+), and (NAG)-(δ-Mur)-(NAG)-(δ-Mur)-(NAG)-(δ-Mur)-(NAG)-(NAM)-Ala-Glu-Dpm-Ala (ε=1.10 ppm for M_0+3H^+) (Figure S3). We have re-constructed cortex PG's polymeric structure based on these fragments by reversing the effect of the mutanolysin enzyme, and found that cortex PG has NAMs in between long and repeating δ-Mur and NAG, and that the main peptide stem is Ala-Glu-Dpm-Ala. Additionally, our isolated cortex PG could have a very low cross-linking ratio because only one of the detected hydrolysates shows that a Dpm connects to two Ala and one Glu.

Cortex PG's contribution to spores' water sorption

To quantitatively analyze cortex PG's contribution to spores' water sorption, we weighted cortex PG's water uptake by cortex PG's volume ratio in a spore (Figure 2e). To correlate the mass information of cortex PG and spores from DVS measurements to their volumetric information from the 3D mapping, we used a density relationship to estimate spore and cortex PG's densities at various relative humidity (RH),[3] given by

$$\rho(RH) = x_{\mathrm{nw}}(RH) \cdot \rho_{\mathrm{nw}}^{\mathrm{o}} + x_{\mathrm{w}}(RH) \cdot \rho_{\mathrm{w}}^{\mathrm{o}} \text{ — (S2)}$$

where ρ is the density of cortex PG or spores, x_{nw} and x_{w} are mass ratios of non-water component and water in cortex PG or spores, $\rho_{\mathrm{nw}}^{\mathrm{o}}$ (1300 kg m^{-3}) and $\rho_{\mathrm{w}}^{\mathrm{o}}$ (997 kg m^{-3}) are densities of non-water components and water, respectively. The volume change of cortex PG and spores at various RH levels was obtained by

$$V = M_{\mathrm{nw}}^{\mathrm{o}}(1 + x_{\mathrm{w}})/\rho \text{ — (S3)}$$

where V is the volume of spore or cortex PG and $M_{\mathrm{nw}}^{\mathrm{o}}$ is the mass of non-water components (dried cortex PG or spores). The volumes of cortex PG under 90% RH were scaled to 52.4% (obtained from the 3D mapping, Figure S1e) of a spore's volume. The mass of the non-PG component in a spore was calculated by using a mass balance equation, given by

$$M_{\mathrm{non-PG}} = M_{\mathrm{spore}} - M_{\mathrm{PG}} \text{ — (S4)}$$

where M_{spore}, M_{PG} and $M_{\mathrm{non-PG}}$ are the masses of spores, cortex PG, and non-PG components at various RH levels, respectively. The contributions of cortex PG and non-PG components on spore's water sorption at various RHs were obtained by comparing to their masses at 5% RH (Figure 2i). We also calculated the ratio of water in cortex PG to water in spores at each RH level (Figure S5).

Young's modulus of PG

PG's Young's modulus was characterized by performing the AFM nano-indentation using a probe (NCHV, Bruker) with a spring constant of 42 N m^{-1} and a tip radius of 16 nm. During the nano-indentation, we controlled the RH to be stabilized at various levels. The resulting force *vs*. displacement curves (Figure S8a-d) were analyzed using the Hertz model, given by

$$(F)^{2/3} = (\frac{4}{3}\frac{E_{\mathrm{PG}}}{(1-v)^2}\sqrt{\mathrm{R_{tip}}})^{2/3} d \text{ — (S5)}$$

where F is the indentation force, E_{PG} is the Young's modulus, v is the Poisson's ratio (0.32),[4,5] $\mathrm{R_{tip}}$ is the tip radius, and d is the indentation depth. PG's Young's moduli (stiffness) at RHs from 5% to 90% are shown in Figure S8e. Before the measurements, AFM cantilever's spring constant was calibrated by using a thermal tune function (Bruker), the deflection sensitivity was calibrated by performing indentations on a hard silicon surface, and the tip radius was calibrated using a standard calibration kit (Bruker). PG's Young's moduli were further characterized by performing nano-indentations using AFM tips with various tip radii and spring constants, and they all show similar values.

Spring constant calibration of AFM cantilevers

The spring constant (k) of the AFM probe (LRCH-250, Team Nanotec) used in the energy/power density measurement was calibrated by a pre-calibrated cantilever (CLFC-NOBO, Bruker) with a spring constant ($\mathrm{k_{ref}}$) of 10.4 N/m. We fixed the CLFC cantilever on a sample stage and performed nano-indentations using LRCH-250 at the end of the CLFC cantilever for five times to obtain an averaged deflection sensitivity (S_{ref}). Subsequently, the spring constant of the LRCH-250 cantilever was calculated by using

$$k = \mathrm{k_{ref}}(\frac{S_{\mathrm{ref}}}{S_{\mathrm{hard}}} - 1) \text{ — (S6)}$$

where S_{hard} is the deflection sensitivity of the LRCH-250 cantilever on a hard silicon surface.

Energy/power densities of PG

PG's energy density is calculated by dividing PG's work done during a thermodynamic cycle by the volume that contributes to the work.[4] Because the spherical shape of the AFM tip (R_{tip}=377 nm, Figure S10a) leads to a non-uniform pressure distribution (Figure S10b), the maximum contact pressure locates at the contact center. In this case, a large portion of the volume under the contact area cannot reach the limit of its actuation pressure and work. Thus, PG's energy density would be largely underestimated if we directly use the entire volume under the spherical indenter for calculations (~12.6 MJ m^{-3}, calculated by dividing the measured work to the entire volume under the contact area). Therefore, we compared the spherical indenter used in our experiment with a flat indenter, which can lead to a uniform pressure distribution (Figure S10c). By equating the indentation depth and mechanical work between these two indentation scenarios, we calculated the effective volume (V_{eff}) that uniformly contributes to the work. The effective volume can be then used to evaluate PG's energy density.

PG's energy density ($E_{\mathrm{\rho}}$) was calculated by

$$E_{\mathrm{\rho}} = \frac{W_{\mathrm{m}}}{V_{\mathrm{eff}}} \text{ — (S7)}$$

where W_{m} is the experimentally measured work and $V_{\mathrm{eff}} = A_{\mathrm{eff}} T_{\mathrm{PG}}$ — (S8), where T_{PG} is PG's thickness and A_{eff} is the effective contact area. To find the effective contact area (A_{eff}), we approximated the indentation (S-P contact) (Figure S10b) to a plane-plane (P-P) contact (Figure S10c) by equating the indentation depths and mechanical works of these two indenting conditions. In the S-P contact, the total work ($W_{\mathrm{total_{S-P}}}$) from PG's deformation is given by

$$W_{\mathrm{total_{S-P}}} = W_{\mathrm{t_{S-P}}} + W_{\mathrm{b_{S-P}}} \text{ — (S9)}$$

where $W_{\mathrm{t_{S-P}}}$ is the work on PG's top surface, given by $W_{\mathrm{t_{S-P}}} = \int_0^{h_{\mathrm{t}}} \frac{4}{3} E_{\mathrm{PG}} \mathrm{R_{tip}}^{1/2} h^{3/2} \mathrm{d}h$ — (S10), where E_{PG} is the Young's modulus of PG, R_{tip} is the tip radius, and h_t is the top indentation depth, h is the height of PG, and $W_{\mathrm{b_{S-P}}}$ is the work on PG's bottom surface, given by $W_{\mathrm{b_{S-P}}} = \int_0^{h_{\mathrm{b}}} E_{\mathrm{PG}} A_b h / T_{\mathrm{PG}} \mathrm{d}h$ — (S11), where h_b is the bottom indentation depth and A_b is the PG's bottom contact area, which is assumed as twice of the top contact area, $A_{\mathrm{b}} = 2\pi R_{\mathrm{tip}} h_{\mathrm{t}}$ — (S12), to avoid overestimation. In the P-P contact, top and bottom indentation depths are equivalent, and hence the total work ($W_{\mathrm{total_{P-P}}}$) done is given by

$$W_{\text{total}_{\text{P-P}}} = \frac{E_{\text{PG}} A_{\text{eff}} {h_{\text{p-p}}}^2}{T_{\text{PG}}} \text{ — (S13)}$$

where $h_{\text{p-p}}$ is the indentation depth in P-P contact. The effective contact area (A_{eff}) was then determined by equating the top indentation depth in S-P contact to that in P-P contact, $h_{\text{t}} = h_{\text{p-p}}$ — (S14) and equating the total work of S-P contact to that of P-P contact. Note that the Young's modulus of PG (E_{PG}) is canceled out when equating the total work of the two contact scenarios. The parameters used to obtain the effective contact area include the tip radius (R_{tip}), the total indentation depth ($h_{\text{m}} = h_{\text{t}} + h_{\text{b}}$ — (S15)), the thickness of PG (T_{PG}), the tip radius (R_{tip}), and the mechanical work done within a thermodynamic cycle (W_{m}), which are all experimentally determined. Note that PG's energy/power densities are underestimated since only PG's actuation in height is considered.

Estimation of nanoconfined water's viscosity

The viscosity of nanoconfined water was estimated by using poroelasticity theory and Darcy's law of diffusion suggested by ref [6]. The experimental data used for the estimation include PG's WR relaxation time constants, WR strain, Young's modulus, and water sorption isotherms. By considering PG as a spherical structure, nanoconfined water's poroelastic diffusion coefficient (D_{p}) can be calculated by [7]

$$\tau = \frac{{R_{\text{PG}}}^2}{\pi^2 D_{\text{p}}} \text{ — (S16)}$$

where τ is the relaxation time constant (see Experimental Section/Methods) and R_{PG} is the radius of PG. Subsequently, nanoconfined water's viscosity was estimated by [6]

$$D_{\text{p}} = \frac{(1-\nu_s)}{(1-2\nu_s)(1+\nu_s)} \frac{\varphi}{4\kappa} \frac{E_{\text{PG}}\xi^2}{\mu_{\text{w}}} \text{ — (S17)}$$

where ν_s is the Poisson ratio of dehydrated PG, ξ is the pore radius, μ_{w} is the viscosity of nanoconfined water, κ is a parameter that accounts for irregularity, interconnectivity, and tortuosity of the pores, E_{PG} is PG's Young's modulus, and φ is the porosity of PG that is given by

$$\varphi = V_{\text{water}}/(V_{\text{water}} + V_{\text{PG}}) \text{ — (S18)}$$

where V_{water} is the volume of water inside PG, V_{PG} is the volume of PG's solid network. In our system, the Poisson ratio (ν_s) is estimated to be 0.32,[5] and the geometry constant (κ) is estimated to be 4.[6] For PG's radius (R_{PG}), Young's modulus (E_{PG}), porosity (φ), and pore radius (ξ), we used their values at 0.5 τ calculated by

$$\frac{\Gamma(0.5\tau)-\Gamma_{\mathrm{h}}}{\Gamma_{\mathrm{d}}-\Gamma_{\mathrm{h}}} = e^{-0.5} \text{ — (S19)}$$

where $\Gamma(0.5\ \tau)$ is the property value at 0.5 τ, and Γ_{h} and Γ_{d} are property values when PG is hydrated and dehydrated, respectively. Using this method, PG's radius was calculated to be 78.0 nm, the Young's modulus was calculated to be 3.42 GPa, and the porosity was calculated to be 0.0045. The pore radius of PG was estimated to be 0.199 nm using the average pore radius of PG in liquids (11.3 nm)[8] and PG's WR strain. Using these values, the poroelastic diffusion coefficient and viscosity of nanoconfined water were calculated to be 3.32×10^{-15} m^2 s^{-1} and 16.4 Pa·s, respectively.

Young's moduli of PG/adhesive composites, adhesive films, polymer films, and PG/adhesive-coated polymer films

Young's moduli of standing alone PG/adhesive composites and adhesive films: We characterized the Young's moduli of standing alone PG/adhesive composites and adhesive films at 10% and 90% RH using a tensile tester (ElectroForce 5500, TA Instruments), which is equipped with a RH control system. PG/adhesive composite samples were prepared by depositing a 20 µL solution, consisting of 29.3 mg mL^{-1} of PG and 10.7 mg mL^{-1} of adhesive, on a 3 mm × 15 mm Mylar film. The solution was allowed to dry under ambient conditions to form a 14 µm thick PG/adhesive composite film. The PG/adhesive composite film was then peeled off from the Mylar film for the tensile test. The adhesive film samples were prepared by peeling off a 160 µm thick dried adhesive film deposited on Mylar films. During the tensile test, each film's strain (ε) and stress (σ) were collected and fitted by

$$\sigma = E\varepsilon \text{ — (S20)}$$

to obtain their Young's moduli (E) (Figure S13). The Young's modulus of PG within the composites was then estimated by

$$E_{\mathrm{PG}} = (E_{\mathrm{c}} - E_{\mathrm{ad}} f_{\mathrm{ad}})/f_{\mathrm{PG}} \text{ — (S21)}$$

where E_{PG}, E_{c}, and E_{ad} are Young's moduli of PG, composite and adhesive film, respectively, and f_{ad} and f_{PG} are the volume fractions of adhesive and PG in the composite. Using equation S21, we estimated Young's modulus of PG to be 1.77 GPa at 5% RH and 0.63 GPa at 90% RH.

Young's moduli of Mylar, OOMOO 25 silicone, and PDMS films: Young's moduli of Mylar, OOMOO 25 silicone, and PDMS films were characterized by using the same tensile test method mentioned above (Figure S14). The dimensions of Mylar, OOMOO 25 silicone, and PDMS film

sample are 40 mm × 5 mm × 12 μm, 6 mm × 6 mm × 0.5 mm, and 20 mm × 15 mm × 0.5 mm (length × width × thickness), respectively. Young's moduli of Mylar, OOMOO 25 silicone, and PDMS films are shown in Table S3.

Young's moduli of PG/adhesive-coated Mylar, OOMOO 25 silicone, and PDMS films: Young's moduli of PG/adhesive-coated polymer films were characterized by using the same tensile test method mentioned above (Figure S14). Sample films of 2.5 mm × 3 mm × 0.02 mm Mylar, 12 mm × 6 mm × 0.6 mm OOMOO 25 silicone, and 4 mm × 3 mm × 0.5 mm PDMS with 8 μm thick PG/adhesive composites coated on their surfaces were used for the test. Young's moduli of PG/adhesive-coated Mylar, OOMOO 25 silicone, and PDMS films are shown in Table S3.

Energy densities of PG/adhesive composites

For PG-based bilayer structures, the WR energy of PG/adhesive composites can be estimated from the elastic bending energy of the passive substrates by

$$U_{\mathrm{ext}} = \frac{(E_{\mathrm{s}}I_{\mathrm{s}})L}{2R^2} \text{ — (S22)}$$

where U_{ext} is the elastic bending energy, E_{s} is the Young's modulus of the substrate, I_{s} is the area moment of inertia of the substrate, R is the radius of curvature, and L is the length of the substrate. The area moment of inertia of the substrate (I_{s}) can be given by

$$I_{\mathrm{s}} = \frac{\pi {t_{\mathrm{s}}}^4}{64} + \pi {t_{\mathrm{s}}}^2\left(\frac{t_{\mathrm{s}}}{2} - H\right)^2 \text{ — (S23) for the glass fiber}$$

and $$I_{\mathrm{s}} = \frac{b_{\mathrm{s}}{t_{\mathrm{s}}}^3}{12} + b_{\mathrm{s}}t_{\mathrm{s}}\left(\frac{t_{\mathrm{s}}}{2} - H\right)^2 \text{ — (S24) for Mylar, OOMOO 25 silicone, and PDMS films}$$

where t_{s} is the thickness of the substrate, b_{s} is the width of the substrate, and H is the location of the neutral axis calculated by using the Stoney's equation:[9]

$$H = t_{\mathrm{s}} - \frac{{E_{\mathrm{c}}}^2{t_{\mathrm{c}}}^4 + E_{\mathrm{c}}E_{\mathrm{s}}t_{\mathrm{c}}{t_{\mathrm{s}}}^2(3t_{\mathrm{c}} + 4t_{\mathrm{s}})}{6E_{\mathrm{c}}E_{\mathrm{s}}t_{\mathrm{c}}t_{\mathrm{s}}(t_{\mathrm{c}} + t_{\mathrm{s}})} \text{ — (S25)}$$

where E_{c} is the Young's modulus of the PG/adhesive composites (1.33 GPa, Figure S13) and t_{c} is the thickness of the PG/adhesive composites. The energy density of the PG/adhesive composites is estimated by

$$D = \frac{U_{\mathrm{ext}}}{V_{\mathrm{c}}} \text{ — (S26)}$$

where D is the energy density, V_{c} is the volume of the PG/adhesive composites, calculated by

$$V_c = Lbt_c \text{ — (S27)}$$

where b is the width of the PG/adhesive layer.

Our calculations show that the energy density of the PG/adhesive composites coated on glass fibers is 4.90 MJ m^{-3} (R is 0.22 mm, b is 10.6 µm, t_c is 11.6 µm, t_s is 10.6 µm, and H is calculated as 4.44 µm). PG/adhesive composites coated on Mylar, OOMOO 25 silicone, and PDMS films show energy densities of 0.35 MJ m^{-3}, 0.96 MJ m^{-3}, and 0.88 MJ m^{-3}, respectively.

Preparation of origami structures, soft grippers, and PG-based push and pull actuators

Origami structures: To prepare the origami cube, a 12 µm thick Mylar film was hand-cut into a cubic structure's expanded form, and 2 mm × 5 mm × 1.8-3.3 µm (length × width × thickness) PG/adhesive composites were then coated on five edges, allowing the cube to fully close at ~30% RH (Movie S6).

Soft grippers: The PG-based soft gripper consists of two soft active fingers, each of which was prepared by assembling twenty 6 mm × 15 mm PG/adhesive-coated Mylar films and twenty sets of 3 mm × 6 mm × 25 µm (length × width × thickness) Mylar film spacers into a portable RH-control system (Figure S19). The PG/adhesive-coated Mylar film was prepared by depositing an 8 µL solution, consisting of 58.5 mg mL^{-1} PG and 18.7 mg mL^{-1} adhesive, on a 3 mm × 6 mm area of a 12 µm thick Mylar film. Subsequently, the PG/adhesive solution was allowed to dry to form a 27 µm thick PG/adhesive composite layer.

Push actuators: To fabricate the push actuator, we prepared two wavy-shaped actuators, each of which was made by depositing PG/adhesive composites on alternating sides of a 30 mm × 15 mm × 12 µm (length × width × thickness) Mylar film. We first coated PG/adhesive composites on one side, giving alternately coated and uncoated regions of 2 mm × 15 mm and 4 mm × 15 mm, respectively, along the length direction. Each PG/adhesive composite coating (2 mm × 15 mm × 14 µm) was formed by depositing a 13.3 µL solution, consisting of 29.3 mg mL^{-1} of PG and 10.7 mg mL^{-1} of adhesive. After the solution dried out, we flipped over the Mylar film, and performed the same deposition on the other side. Subsequently, we connected the two wavy-shaped actuators vertically by attaching polystyrene sheets (Fisher Scientific) on the top and bottom sides to form the final configuration (Figure 6f).

Pull actuators: To fabricate the pull actuator, we prepared 19 wavy-shaped actuators, each of which was made by depositing PG/adhesive composites on alternating sides of a 24 mm × 15

mm × 12 μm (length × width × thickness) Mylar film. We first coated PG/adhesive composites on one side, giving alternately coated and uncoated regions of 3 mm × 15 mm along the length direction. Each PG/adhesive composite coating (3 mm × 15 mm × 14 μm) was formed by depositing a 20 μL solution, consisting of 29.3 mg mL^{-1} of PG and 10.7 mg mL^{-1} of adhesive. After the solution dried out, we flipped over the Mylar film, and performed the same deposition on the other side. Subsequently, these 19 wavy-shaped actuators were bundled by screws and bolts (McMaster Carr), and integrated into a portable RH-control system (Figure S20). An RH-control system was used to control the above-mentioned PG-based actuators (Figure 6a, Figure 6h, Figure S19, and Figure S20). The system is made of acrylic chambers (McMaster-Carr) fabricated using a laser cutter (VLS4.60, Universal Laser). The RH control was achieved by connecting the acrylic chambers to 2mm-OD poly-vinyl chloride (PVC) tubing (SMC), which allows delivering dry or humid air to the actuators controlled by solenoid valves (VK332Y, SMC) and LabVIEW programs.

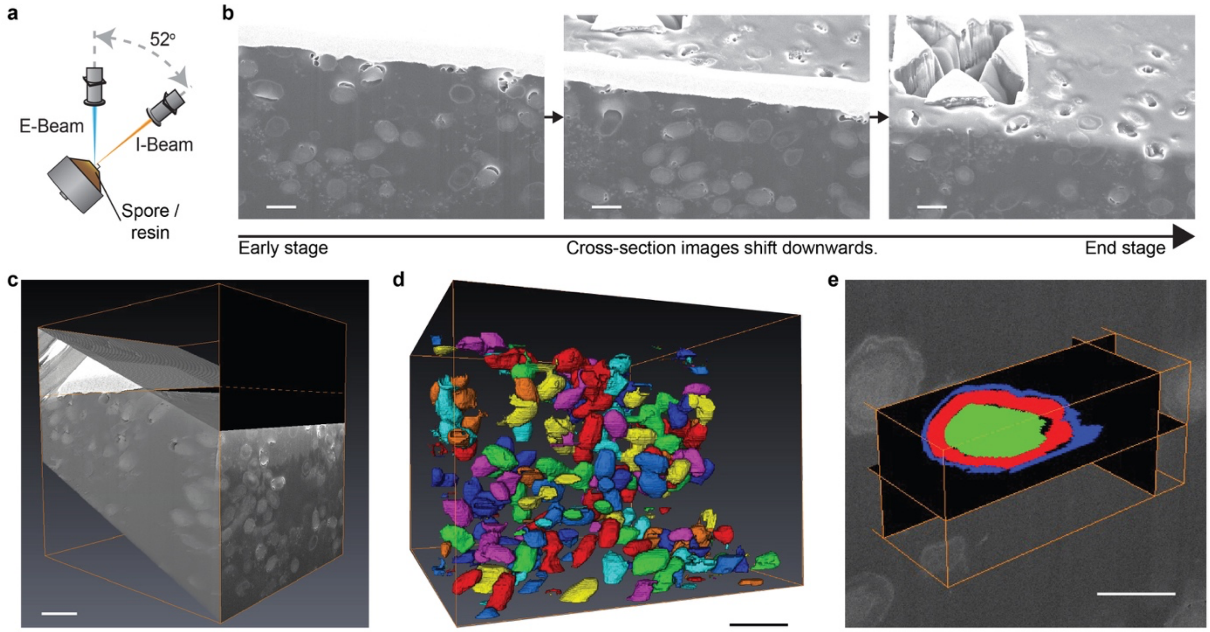

Figure S1. Spores' three-dimensional reconstruction. (a) A spore/resin sample was alternately milled and imaged by an ion beam and an electron beam. (b) Collected serial SEM images shift due to the angle between the ion beam and the electron beam. Scale bar, 1 µm. (c) SEM images were stacked and aligned to form a three-dimensional map of spores. Scale bar, 1 µm. (d) Spores in the resin were identified by the software, Avizo. Scale bar, 1 µm. (e) Spores' components - coats, cortex, and cores - were identified based on the contrast differences. Scale bar, 500 nm.

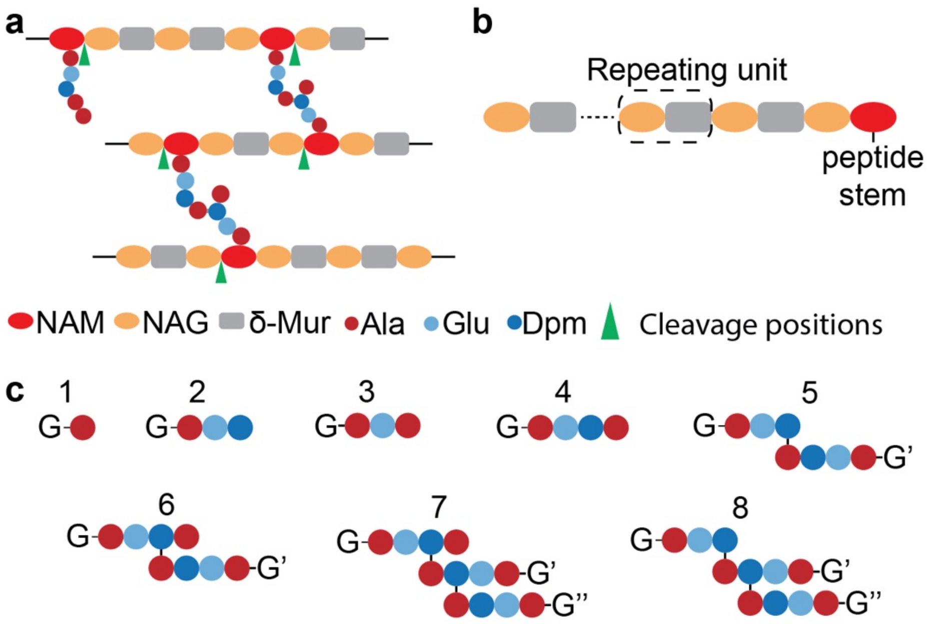

Figure S2. Cortex PG's hydrolysates. (a) The mutanolysin enzyme cleaves the β-(1,4) link between NAM and NAG, leaving various hydrolysates (b). (c) Possible hydrolysates include eight different peptide stems. G, G', and G'' indicate different glycan chains whose structures are shown in (b).

Fragment index:1, EIC 913.9108 ± 0.005, ε=3.22 ppm

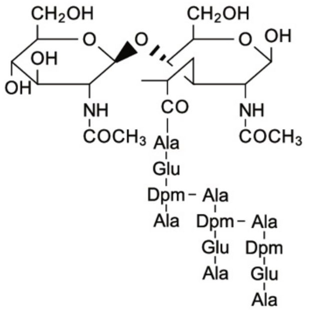

Fragment index:2, EIC 690.7715 ± 0.005, ε=1.58 ppm
Fragment index:2, EIC 679.7799 ± 0.005, ε=2.04 ppm

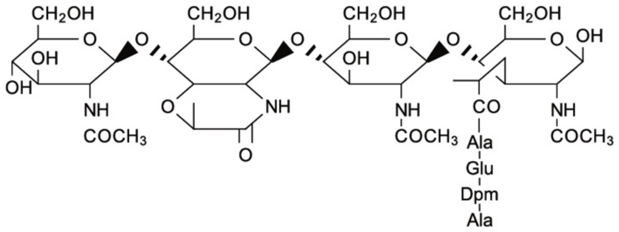

Fragment index:3, EIC 702.7770 ± 0.005, ε=1.99 ppm

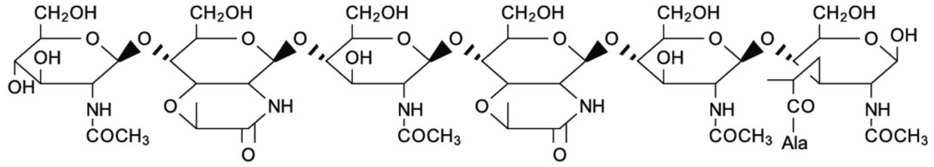

Fragment index:4, EIC 888.8589 ± 0.005, ε=1.77 ppm

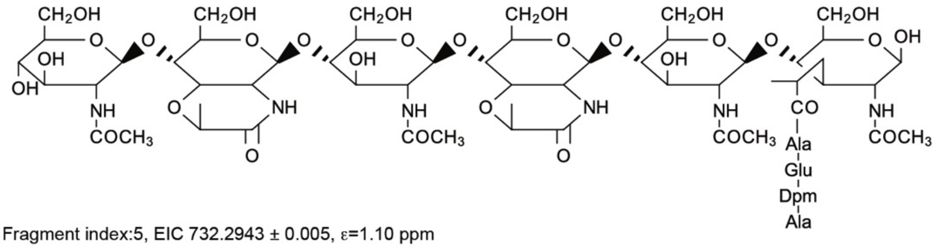

Fragment index:5, EIC 732.2943 ± 0.005, ε=1.10 ppm

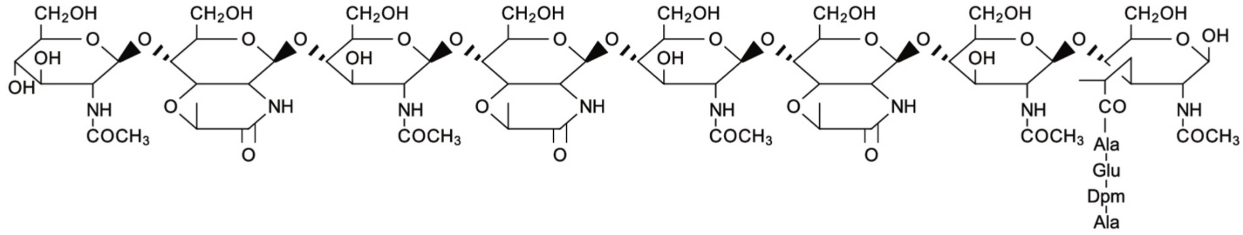

Figure S3. The PG fragments, identified by using the LC-ESI-MS, show that one NAM attaches to repeating NAG and δ-Mur. The peptide stem is mainly composed of Ala-Glu-Dpm-Ala except for fragment #3 where only one Ala is present.

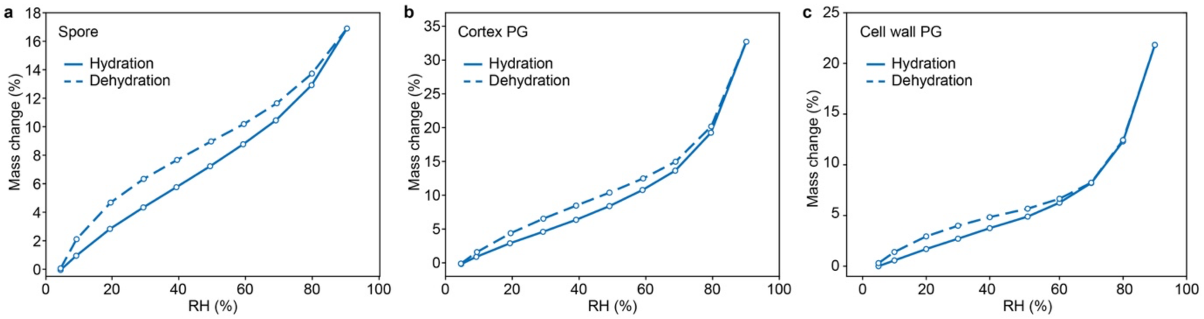

Figure S4. Spore and PGs' water sorption isotherms. The isotherms of *B. subtilis* spores (a), cortex PG (b), and cell wall PG (c) were measured at each RH when the RH is cycled between 5% and 90% RH at 25 ^{o}C. Note that these isotherms are highly repeatable.

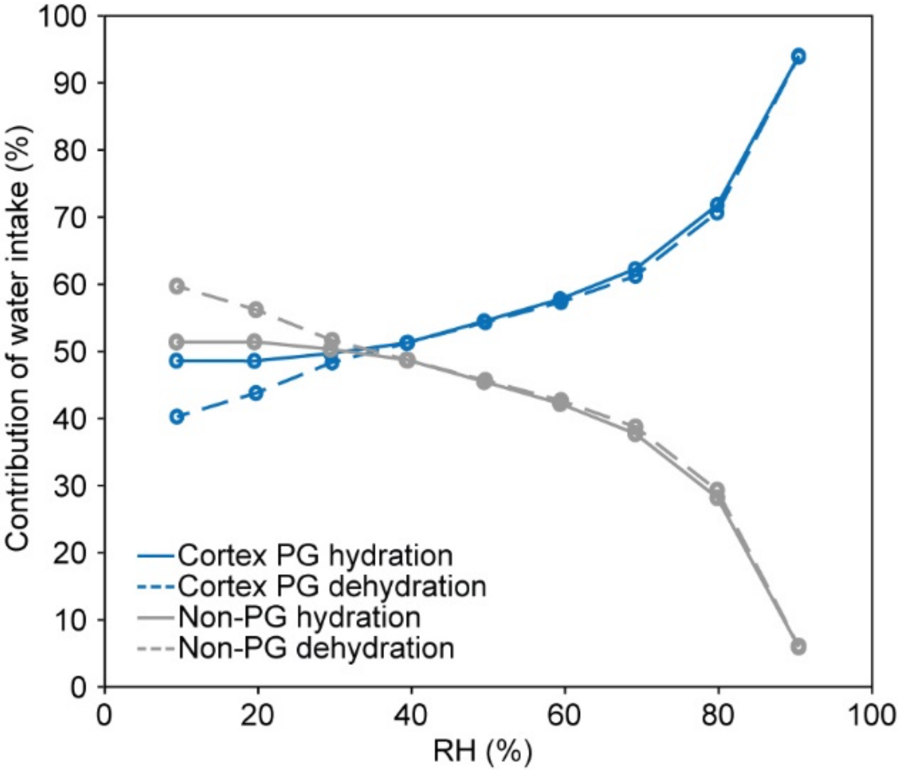

Figure S5. Cortex PG and non-PG's contributions to spores' water sorption. The trends show that cortex PG contributes to ~50% and ~94% of spores' water exchange at 5% and 90% RH, respectively.

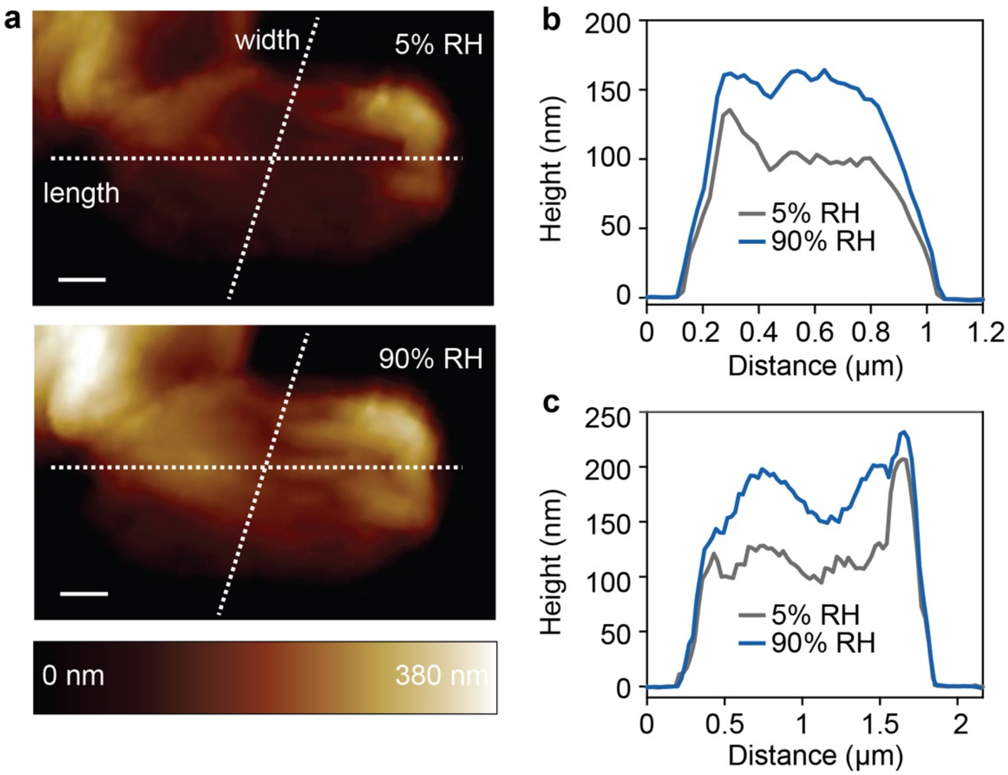

Figure S6. Cortex PG's topographic images at 5% RH and 90% RH. (a) Cortex PG expands when RH is increased from 5% to 90%. Scale bar, 200 nm. (b) Cross-sectional width profiles of cortex PG at 5% RH (grey) and 90% RH (blue). (c) Cross-sectional length profiles of cortex PG at 5% RH (grey) and 90% RH (blue). By analyzing cortex PG's topographic changes, the volume change of cortex PG was estimated to be 65.2%, which is averaged from three measurements with a standard error of 1.17%.

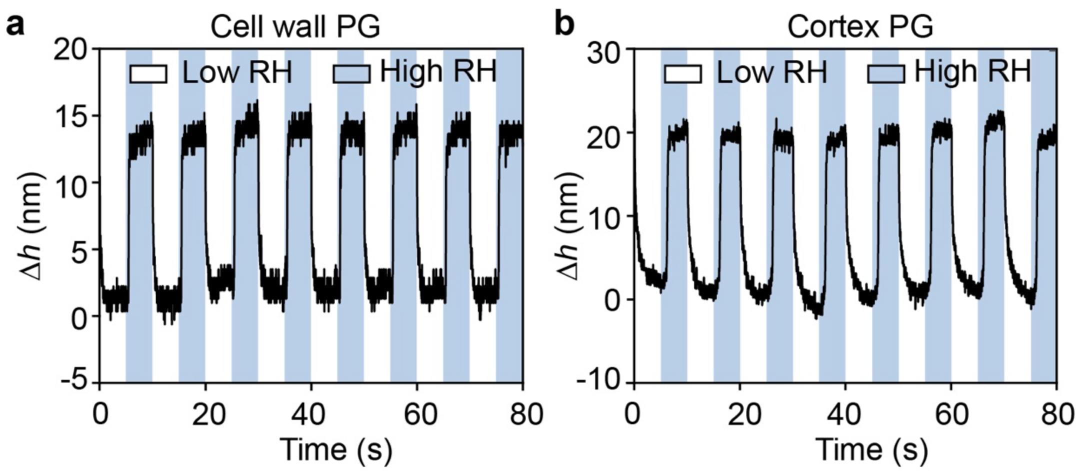

Figure S7. The reversibility of PGs' WR deformation. (a-b) The dynamic height changes of cell wall PG and cortex PG when RH alternates between 5% and 90%. These tests show negligible decays in PGs' WR deformation during 8 cycles with a cycle time of 10 s.

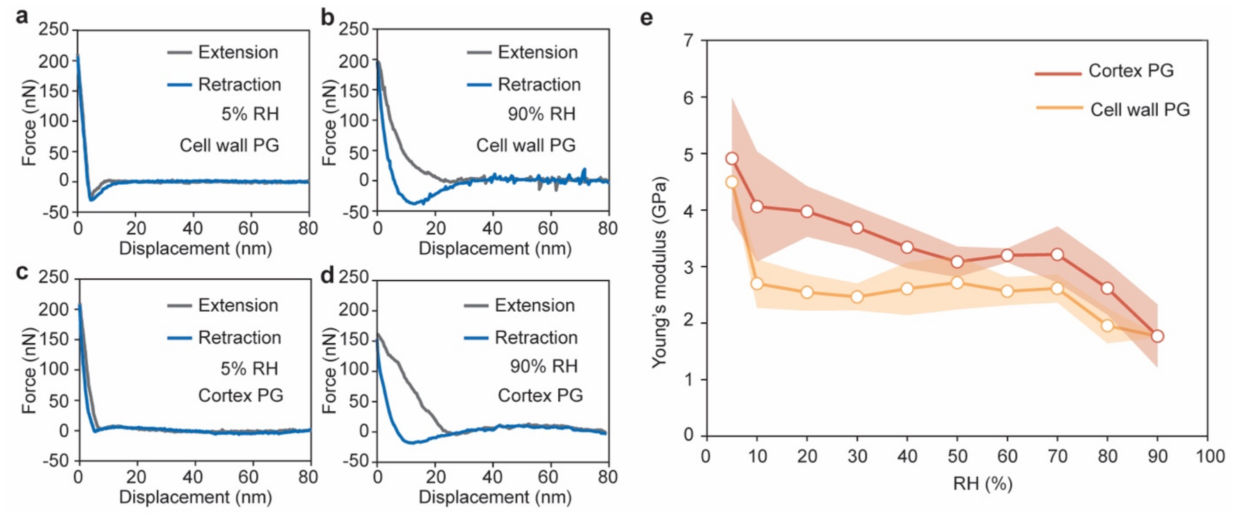

Figure S8. Young's moduli of cell wall PG and cortex PG. The force *vs.* displacement curves of cell wall PG (a-b) and cortex PG (c-d) at 5% and 90% RH, characterized by the AFM indentation. (e) Both cortex PG's and cell wall PG's Young's moduli decrease when the RH increases. Standard errors are calculated from five measurements.

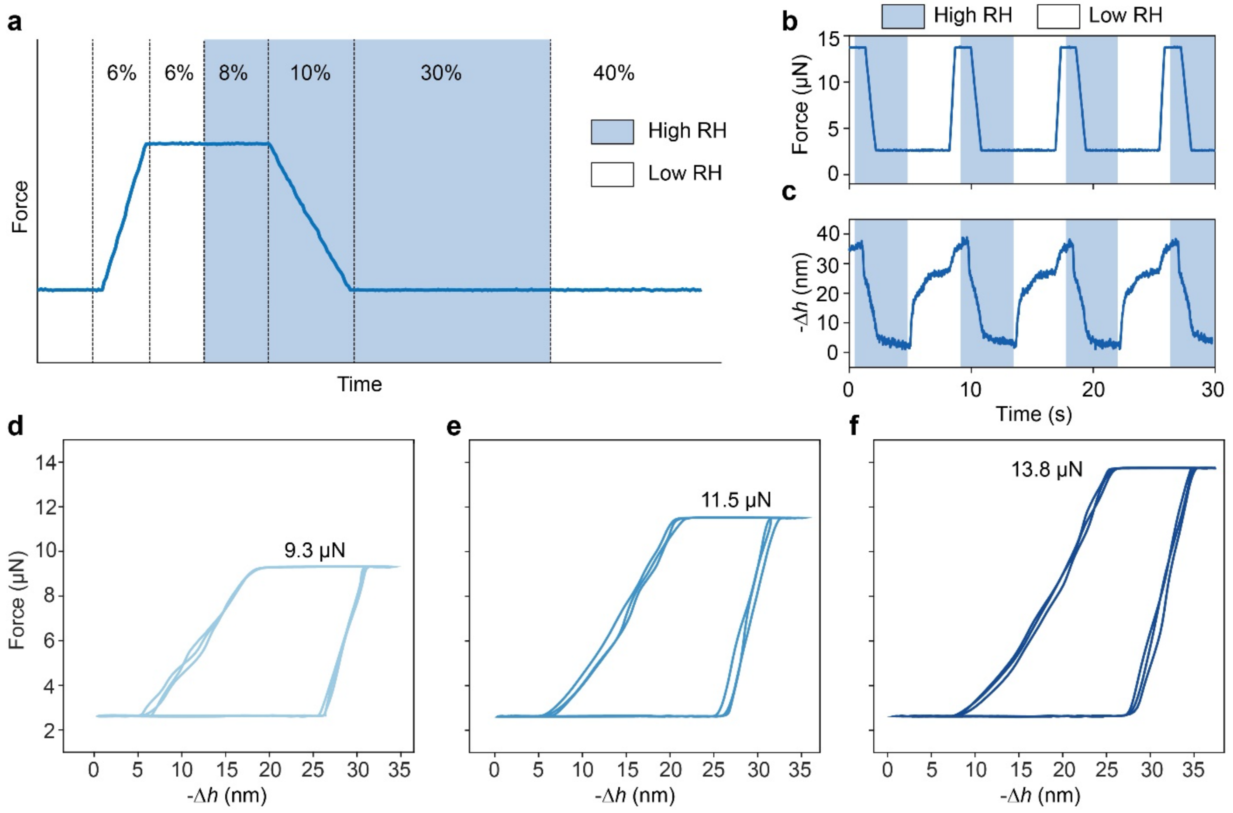

Figure S9. Cortex PG's thermodynamic cycles. (a) The force and the duration of each stage in a thermodynamic cycle for cortex PG's energy density measurements. The total cycle time varies from 8 s to 12 s. (b-c) The applied force (b) and measured -Δh (c) of cortex PG. (d-f) The *F vs.* -Δh curves are highly repeatable with different applied forces (9.3 μN-13.8 μN).

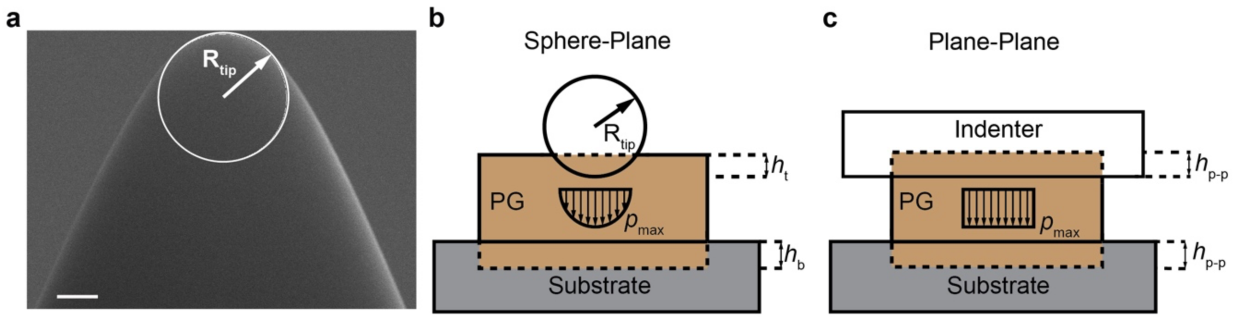

Figure S10. The contact between the AFM tip and PG. (a) A SEM image of the AFM cantilever (LRCH-250) used in energy/power density measurements. Scale bar, 200 nm. (b) The indentation between the AFM cantilever and PG can be estimated as a S-P contact, leading to a non-uniform pressure distribution. (c) The P-P contact model used in PG's energy density calculations.

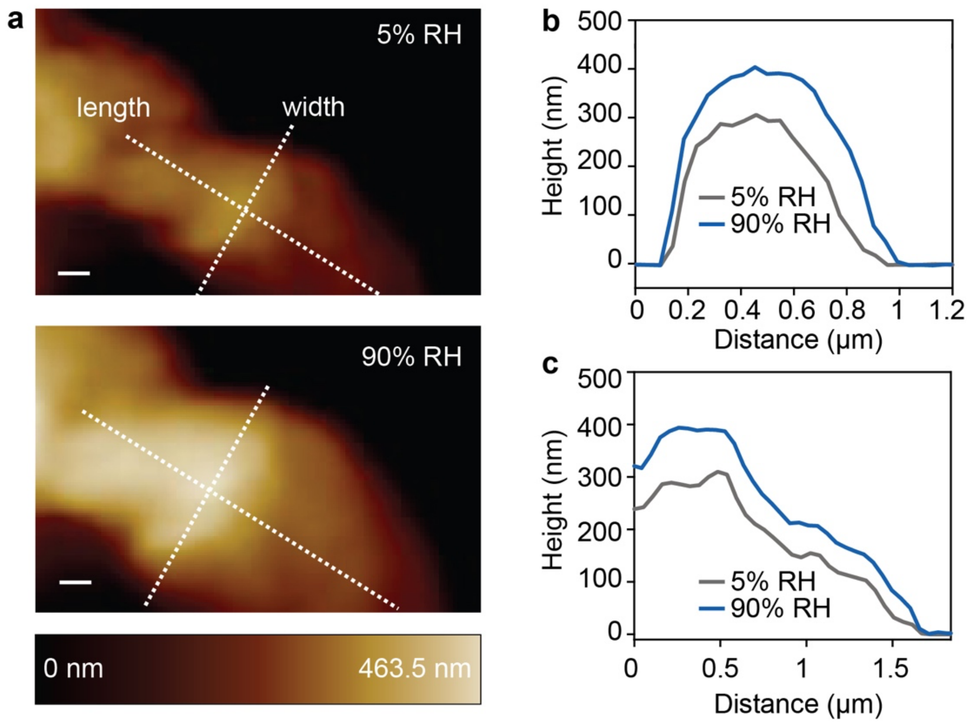

Figure S11. Cell wall PG's topographic images at 5% RH and 90% RH. (a) Cell wall PG expands when RH is increased from 5% to 90%. Scale bar, 100 nm. (b) Cross-sectional width profiles of cell wall PG at 5% RH (grey) and 90% RH (blue). (c) Cross-sectional length profiles of cell wall PG at 5% RH (grey) and 90% RH (blue). By analyzing cell wall PG's topographic changes, the volume change of cell wall PG was estimated to be 45.8%, which is averaged from three measurements with a standard error of 4.45%.

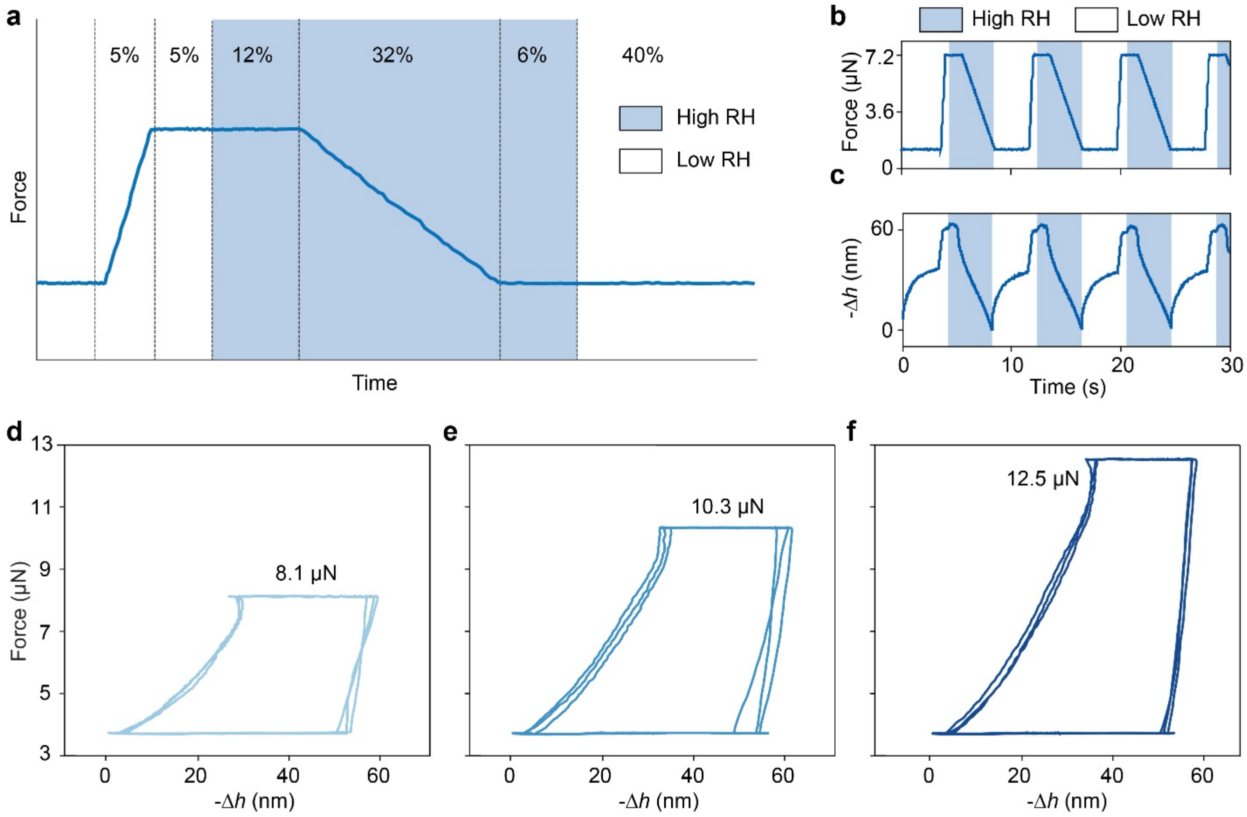

Figure S12. Cell wall PG's thermodynamic cycles. (a) The force and the duration of each stage in a thermodynamic cycle for cell wall PG's energy density measurements. The total cycle time varies from 8 s to 12 s. (b-c) The applied force (d) and measured -Δh (c) of cell wall PG. (d-f) The F *vs.* -Δh curves are highly repeatable with different applied forces (8.1 μN-12.5 μN).

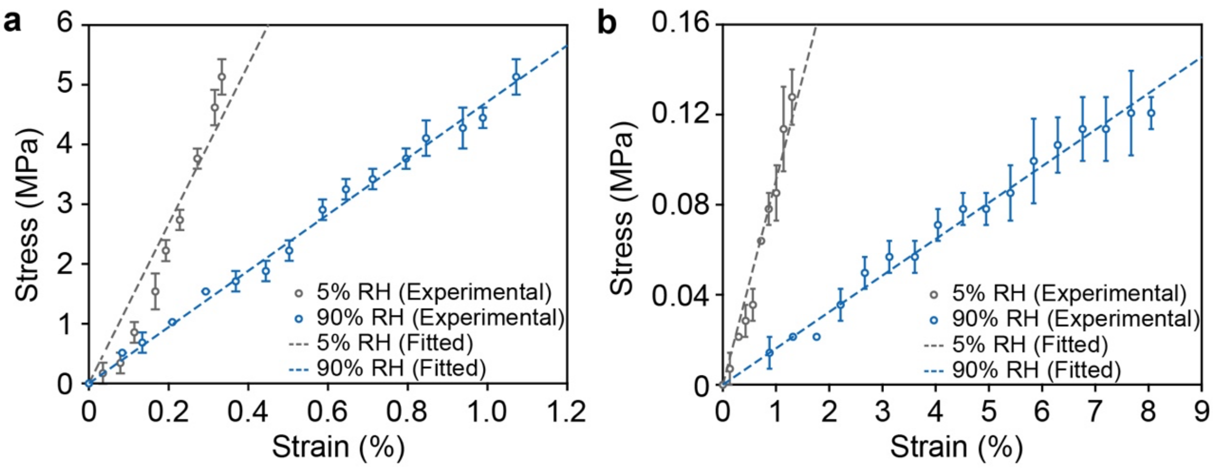

Figure S13. Tensile stress *vs*. strain curves of the PG/adhesive composites and adhesive. The stress *vs*. strain curves of a 3.80 mm × 1.83 mm × 14 μm (length × width × thickness) PG/adhesive composite film (a) and a 5.50 mm × 2.93 mm × 160 μm (length × width × thickness) adhesive film (b) at 5% (grey) and 90% RH (blue). The Young's modulus of the PG/adhesive composite was characterized to be 1.33 GPa at 5% RH and 0.47 GPa at 90% RH, and the Young's modulus of the adhesive film was characterized to be 9.10 MPa at 5% RH and 1.61 MPa at 90% RH. Error bars represent standard errors calculated from three measurements.

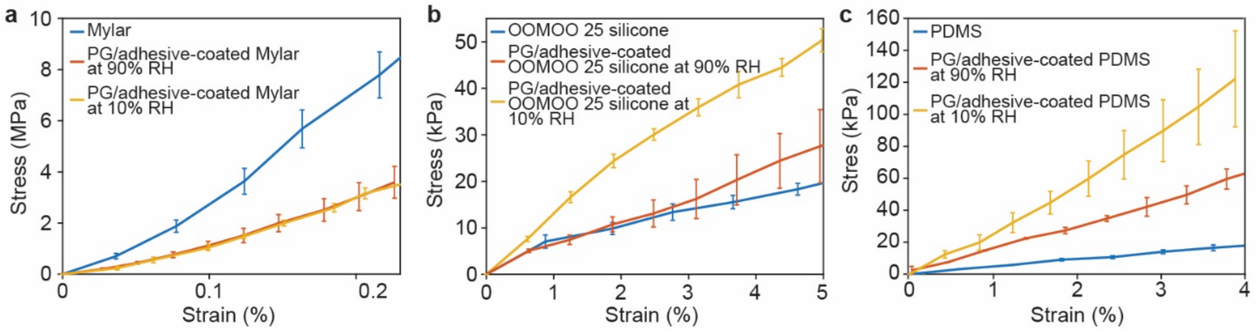

Figure S14. Tensile stress vs. strain of substrate films and PG/adhesive-coated films. (a) Stress *vs*. strain curves of Mylar films (blue) and PG/adhesive-coated Mylar films at 10% RH (yellow) and 90% RH (orange). (b) Stress *vs*. strain curves of OOMOO 25 films (blue) and PG/adhesive-coated OOMOO 25 films at 10% RH (yellow) and 90% RH (orange). (c) Stress *vs*. strain curves of PDMS films (blue) and PG/adhesive-coated PDMS films at 10% RH (yellow) and 90% RH (orange). Error bars represent standard errors calculated from three measurements.

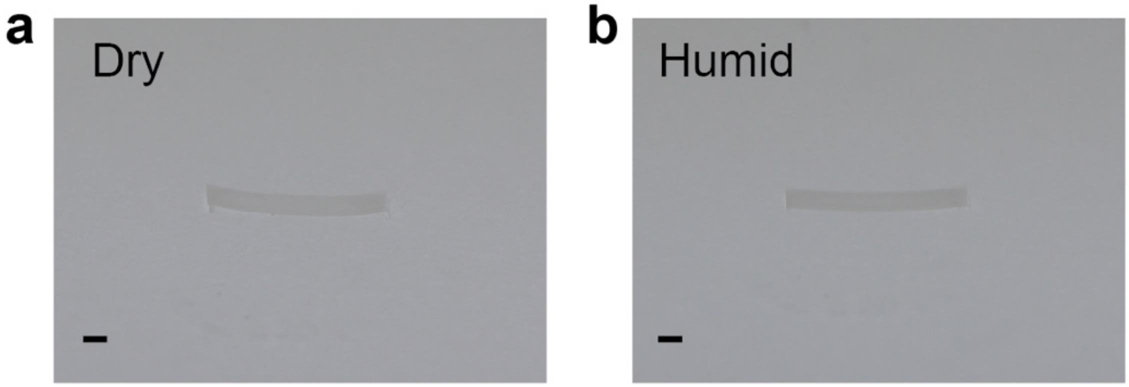

Figure S15. The adhesive shows negligible water-responsiveness. (a-b) An adhesive-coated Mylar film shows negligible WR actuation when RH is changed between 5% and 90%. The WR energy density of the adhesive is estimated to be 3.42×10^{-4} MJ m^{-3}. Scale bar, 1 mm.

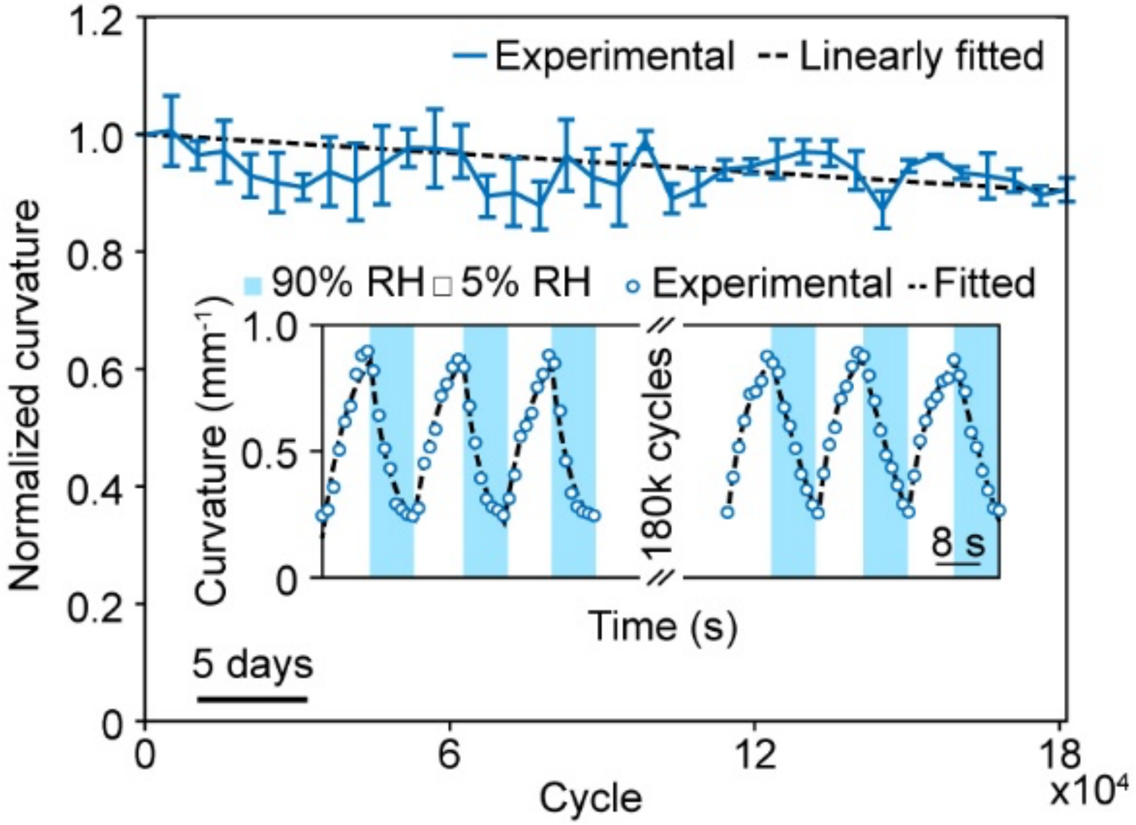

Figure S16. The reversibility of PG/adhesive-coated Mylar films. The normalized curvature characterized at 5% RH for over a month shows that the PG/adhesive-coated Mylar films reversibly bend over 180,000 cycles with a normalized curvature decay rate of 5.17×10^{-7} $cycle^{-1}$. The inset shows that a Mylar film's curvature over time before and after 180,000 cycles. Error bars represent standard errors calculated from three measurements.

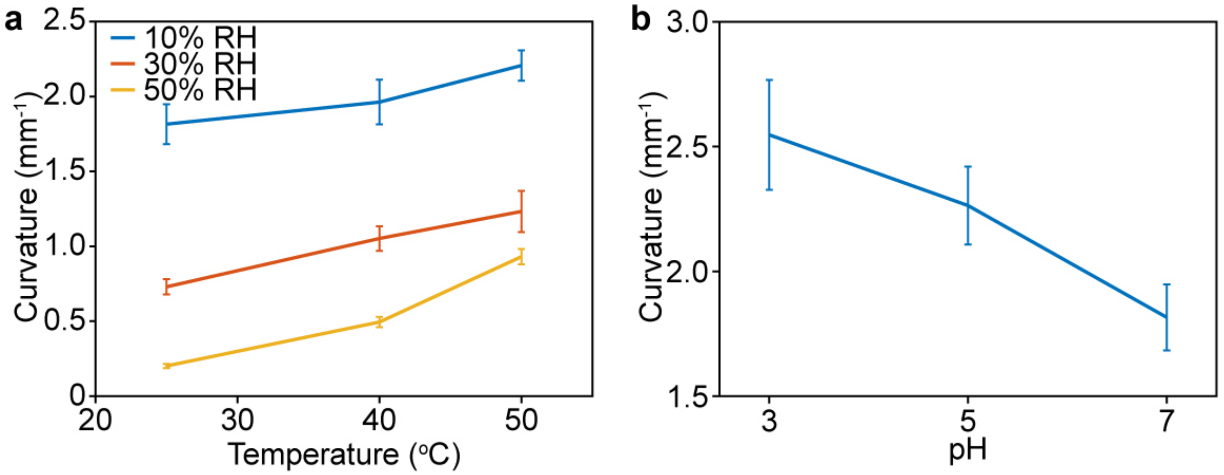

Figure S17. The effect of temperature and pH on the water-responsiveness of PG/adhesive-coated Mylar films. (a) PG/adhesive-coated Mylar films' WR curvatures at different RH increase when temperature increases from 25 ^{o}C to 50 ^{o}C. (b) PG/adhesive-coated Mylar films were immersed into solutions with various pH (pH5 HCl(aq) and pH3 HCl(aq)) for 10 s, and then allowed to dry. These dried films show larger curvatures with increasing pH levels at 10% RH and 25 ^{o}C. Error bars represent standard errors calculated from three measurements.

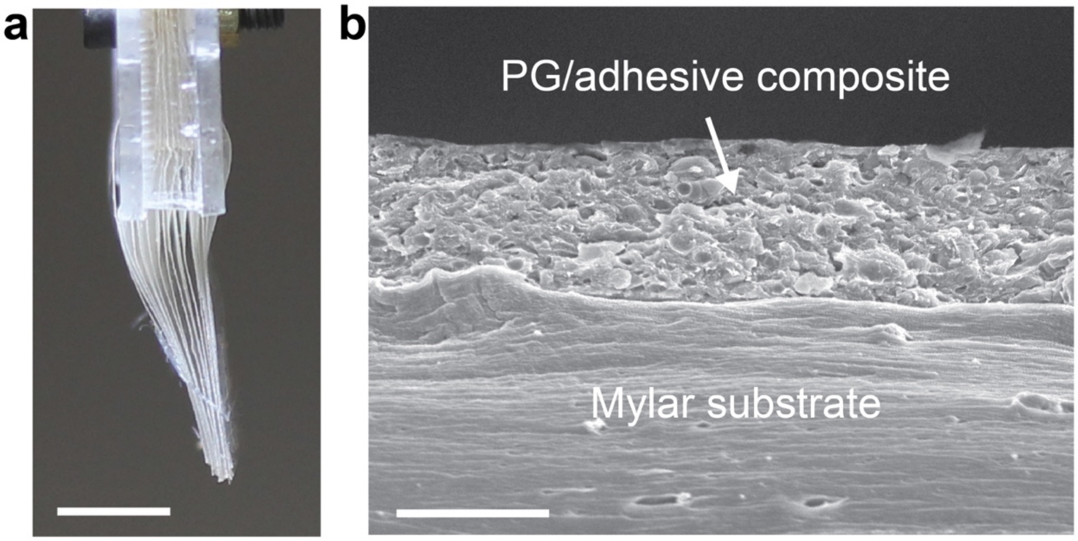

Figure S18. Optical and SEM images of a soft active finger. (a) An optical photo of the side view of an active finger. Scale bar, 5 mm. (b) A cross-sectional SEM of a PG/adhesive-coated Mylar film used in the active finger. Scale bar, 5 μm.

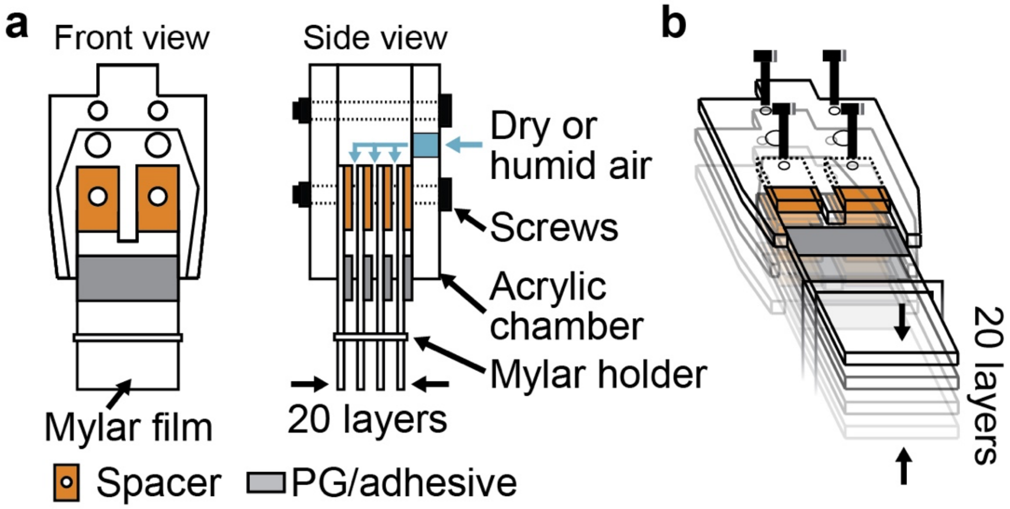

Figure S19. The structure of a soft active finger. (a-b) The schematic of the soft active finger shows that 20 PG/adhesive-coated Mylar films and 20 spacers are alternately stacked. The finger structure includes air channels that allow the delivery of dry or humid air to individual PG/adhesive-coated Mylar films.

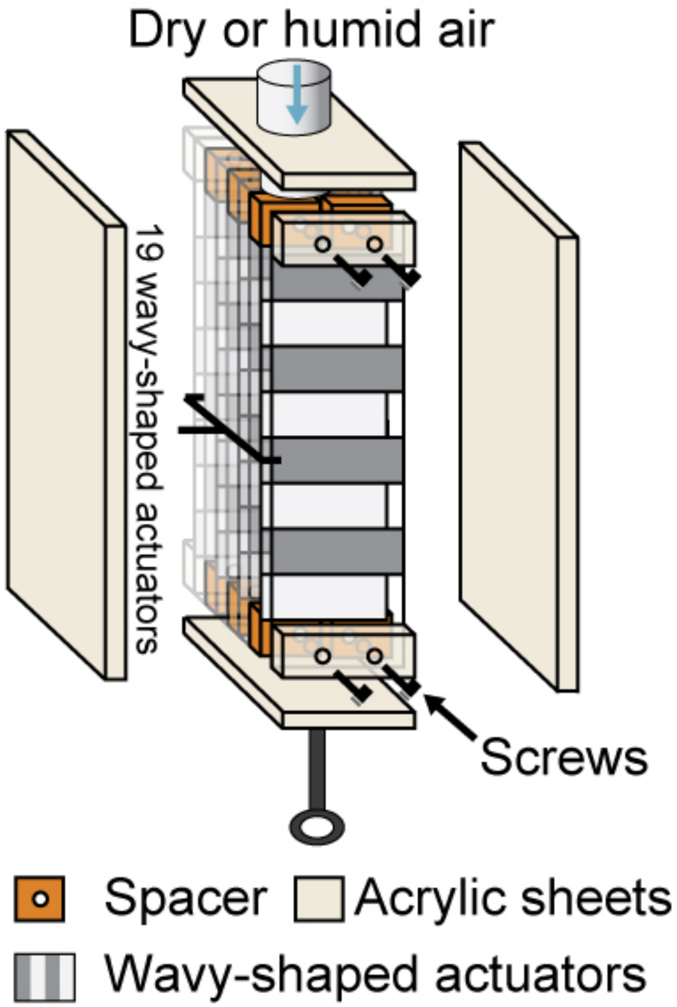

Figure S20. The structure of a PG-based pull actuator. The exploded view of the PG-based pull actuator shows that 19 wavy-shaped actuators and 18 spacers are alternately stacked. These spacers form air channels that allow the delivery of dry or humid air to each wavy-shaped actuator.

Table S1. Standard errors of the water sorption isotherms of spore, cortex PG, and cell wall PG.

RH	Spore standard errors (%)		Cortex PG standard errors (%)		Cell wall PG standard errors (%)	
	Sorption	Desorption	Sorption	Desorption	Sorption	Desorption
**5%**	0.032	0.020	0.018	0.053	0.111	0.016
**10%**	0.029	0.006	0.003	0.068	0.120	0.041
**20%**	0.023	0.009	0.050	0.135	0.123	0.054
**30%**	0.026	0.012	0.098	0.060	0.125	0.042
**40%**	0.027	0.006	0.154	0.097	0.109	0.043
**50%**	0.012	0.003	0.103	0.095	0.071	0.046
**60%**	0.015	0.020	0.111	0.079	0.033	0.040
**70%**	0.006	0.009	0.026	0.081	0.053	0.022
**80%**	0.029	0.033	0.010	0.020	0.155	0.036
**90%**	0.061	0.061	0.161	0.161	0.154	0.154

Table S2. Properties of actuator materials.

Materials	Strain (%)	Stress (MPa)	Power density (kW kg^{-1})	Energy density (kJ kg^{-1})	Efficiency (%)	Ref.
**Cortex PG**	50.1	354.6	5.5	46.1	35.0	
**Cell wall PG**	27.2	205.6	7.0	55.8	66.8	
**Sheath-run artificial muscles**	14.3	~80	1.98	2.35	4.26	[10]
**Strain-programmable artificial muscles**	47.7	~55	0.075	7.42	1.12	[11]
**Shape memory nanocomposite fibers**	NA	NA	~ 0.006	2.77	1.0-1.5	[12]
**Fishing line artificial muscles**	49	140	5.3	2.63	1.32	[13]
**Hybrid carbon nanotube yarn muscles**	5.6	84	27.9	1.36	0.55	[14]
**Electrochemically powered carbon nanotube artificial muscles**	16.5	48	NA	1.12	5.4	[15]
**Fluid-driven origami-inspired artificial muscles**	90	0.6	2	NA	2-5	[16]
**Electrostatic actuators**	124	0.3	0.358	0.070	21	[17]
**Mammalian skeletal muscles**	20	0.35	0.323	0.0386	40	[18,19]
**Insect muscles**	3	NA	0.144	NA	14-16	[20]

Table S3. Young's moduli of Mylar, OOMOO 25 silicone, and PDMS films with and without PG/adhesive composites coated on their surfaces.

**Sample name**	Mylar	PG/adhesive-coated Mylar	OOMOO 25 silicone	PG/adhesive-coated OOMOO 25 silicone	PDMS	PG/adhesive-coated PDMS
**Young's modulus**	3.60 GPa	1.53 GPa (dry) 1.50 GPa (humid)	0.40 MPa	1.01 MPa (dry) 0.56 MPa (humid)	0.43 MPa	3.15 MPa (dry) 1.58 MPa (humid)

Table S4. Advantages and disadvantages of the WR actuators compared to conventional actuators.[21-23]

Actuator types	Advantages	Disadvantages
**Water-responsive materials**	High stress, strain, energy density, power density, and efficiency.	Difficult to control. No existing ready-to-use systems.
**Electricity**	Good strain, stress, efficiency, and high energy density, simple in mechanism and construction, low cost.	High voltage and fields are required.
**Heat**	High stress, strain, and energy density, flexible.	Difficult to control, low efficiency, and short life cycle.
**Magnetism**	Good stress and strain.	Bulky magnets are required, high cost.
**Chemical reaction**	Good strain, stress, and energy density.	Complicated systems.
**Hydraulic**	Fast, high strain, and stress.	Bulky operating method, difficult to control, low efficiency.

Movie S1. 3D reconstruction and segmentation of spores.

Movie S2. A PG/adhesive-coated glass fiber bends and straightens in response to RH changes.

Movie S3. A PG/adhesive-coated Mylar film bends and straightens in response to RH changes.

Movie S4. A PG/adhesive-coated OOMOO 25 silicone film bends and straightens in response to RH changes.

Movie S5. A PG/adhesive-coated PDMS film bends and straightens in response to RH changes.

Movie S6. A precut Mylar film with patterned PG/adhesive composites self-folds into a cubic structure when exposed to a dry environment.

Movie S7. A PG-based finger bends and strengthens controlled by a portable RH-control system.

Movie S8. A PG-based soft gripper grasps a pencil.

Movie S9. A PG-based soft gripper grasps a screwdriver bit.

Movie S10. A PG-based push actuator reversibly raises a screwdriver bit.

Movie S11. A PG-based pull actuator reversibly lifts a 50-gram weight.

Movie S12. A PG-based pull actuator reversibly lifts a 200-gram weight.

References

[1] A. Atrih, G. Bacher, G. Allmaier, M. P. Williamson, S. J. Foster, *J. Bacteriol.* **1999**, *181*, 3956.

[2] A. Atrih, P. Zöllner, G. Allmaier, S. J. Foster, *J. Bacteriol.* **1996**, *178*, 6173.

[3] S. M. Knudsen, N. Cermak, F. Feijó Delgado, B. Setlow, P. Setlow, S. R. Manalis, *J. Bacteriol.* **2016**, *198*, 168.

[4] X. Chen, L. Mahadevan, A. Driks, O. Sahin, *Nat. Nanotechnol.* **2014**, *9*, 137.

[5] J. C. Gumbart, M. Beeby, G. J. Jensen, B. Roux, *PLoS Comput. Biol.* **2014**, *10*, e1003475.

[6] E. Moeendarbary, L. Valon, M. Fritzsche, A. R. Harris, D. A. Moulding, A. J. Thrasher, E. Stride, L. Mahadevan, G. T. Charras, *Nat. Mater.* **2013**, *12*, 253.

[7] E. Detournay, A. H. D. Cheng, in *Analysis and Design Methods*, (Ed: C. Fairhurst), Pergamon, Oxford **1993**.

[8] L. Pasquina-Lemonche, J. Burns, R. D. Turner, S. Kumar, R. Tank, N. Mullin, J. S. Wilson, B. Chakrabarti, P. A. Bullough, S. J. Foster, J. K. Hobbs, *Nature* **2020**, *582*, 294.

[9] G. G. Stoney, C. A. Parsons, *Proc. R. Soc. Lond. A* **1909**, *82*, 172.

[10] J. Mu, M. Jung de Andrade, S. Fang, X. Wang, E. Gao, N. Li, S. H. Kim, H. Wang, C. Hou, Q. Zhang, M. Zhu, D. Qian, H. Lu, D. Kongahage, S. Talebian, J. Foroughi, G. Spinks, H. Kim, T. H. Ware, H. J. Sim, D. Y. Lee, Y. Jang, S. J. Kim, R. H. Baughman, *Science* **2019**, *365*, 150.

[11] M. Kanik, S. Orguc, G. Varnavides, J. Kim, T. Benavides, D. Gonzalez, T. Akintilo, C. C. Tasan, A. P. Chandrakasan, Y. Fink, P. Anikeeva, *Science* **2019**, *365*, 145.

[12] J. Yuan, W. Neri, C. Zakri, P. Merzeau, K. Kratz, A. Lendlein, P. Poulin, *Science* **2019**, *365*, 155.

[13] C. S. Haines, M. D. Lima, N. Li, G. M. Spinks, J. Foroughi, J. D. W. Madden, S. H. Kim, S. Fang, M. Jung de Andrade, F. Göktepe, Ö. Göktepe, S. M. Mirvakili, S. Naficy, X. Lepró, J. Oh, M. E. Kozlov, S. J. Kim, X. Xu, B. J. Swedlove, G. G. Wallace, R. H. Baughman, *Science* **2014**, *343*, 868.

[14] M. D. Lima, N. Li, M. J. de Andrade, S. L. Fang, J. Oh, G. M. Spinks, M. E. Kozlov, C. S. Haines, D. Suh, J. Foroughi, S. J. Kim, Y. S. Chen, T. Ware, M. K. Shin, L. D. Machado, A.

F. Fonseca, J. D. W. Madden, W. E. Voit, D. S. Galvao, R. H. Baughman, *Science* **2012**, *338*, 928.

[15] J. A. Lee, N. Li, C. S. Haines, K. J. Kim, X. Lepró, R. Ovalle-Robles, S. J. Kim, R. H. Baughman, *Adv. Mater.* **2017**, *29*, 1700870.

[16] S. Li, D. M. Vogt, D. Rus, R. J. Wood, *Proc. Natl. Acad. Sci. U.S.A* **2017**, *114*, 13132.

[17] E. Acome, S. K. Mitchell, T. G. Morrissey, M. B. Emmett, C. Benjamin, M. King, M. Radakovitz, C. Keplinger, *Science* **2018**, *359*, 61.

[18] J. D. W. Madden, N. A. Vandesteeg, P. A. Anquetil, P. G. A. Madden, A. Takshi, R. Z. Pytel, S. R. Lafontaine, P. A. Wieringa, I. W. Hunter, *IEEE J. Oceanic Eng.* **2004**, *29*, 706.

[19] R. K. Josephson, *Annu. Rev. Physiol.* **1993**, *55*, 527.

[20] R. K. Josephson, J. G. Malamud, D. R. Stokes, *J. Exp. Biol.* **2001**, *204*, 4125.

[21] A. Miriyev, K. Stack, H. Lipson, *Nat. Commun.* **2017**, *8*, 596.

[22] D. Kongahage, J. Foroughi, *Fibers* **2019**, *7*, 21.

[23] Y. Park, X. Chen, *J. Mater. Chem. A* **2020**, *8*, 15227.
